# Army liposome formulation containing QS-21 render human monocyte-derived macrophages less permissive to HIV-1 infection by upregulating APOBEC3A

**DOI:** 10.1038/s41598-022-11230-8

**Published:** 2022-05-09

**Authors:** Ousman Jobe, Jiae Kim, Daniel O. Pinto, Zuzana Villar, Tiffany Hewitt, Elizabeth H. Duncan, Alexander Anderson, Neelakshi Gohain, Hua Gong, Courtney Tucker, Carl R. Alving, Gary R. Matyas, Elke Bergmann-Leitner, Mangala Rao

**Affiliations:** 1 Henry M. Jackson Foundation for the Advancement of Military Medicine, U.S. Military HIV Research Program, Bethesda, MD USA; 2 Laboratory of Adjuvant and Antigen Research, U.S. Military HIV Research Program, Walter Reed Army Institute of Research, Silver Spring, MD USA; 3 Immunology Core, Biologics Research and Development, Walter Reed Army Institute of Research, Silver Spring, MD USA; 4 Smith and Nephew, Columbia, MD USA; 5 The Catholic University of America, Washington, DC USA

**Keywords:** Immunology, Vaccines, Adjuvants

## Abstract

Monocyte-derived macrophages (MDM) are highly permissive to HIV-1 infection potentially due to the downregulation of innate factors during the differentiation process. The environmental milieu and innate anti-viral factors which are modulated during macrophage differentiation, have been associated with their increased permissiveness to HIV-1 infection. Here, we demonstrate that the Army Liposome Formulation containing MPLA, and QS-21 (ALFQ) activated MDM that are normally permissive to HIV-1 infection to generate a proinflammatory environment and upregulated anti-viral factors notably APOBEC3A. Induction of APOBEC3A by ALFQ decreased permissiveness to HIV-1 infection, while knockdown of APOBEC3A with APOBEC3AsiRNA resulted in a significant loss in the restriction of HIV-1 infectivity. The liposome formulation ALF55, with identical lipid composition but lacking QS-21 had no effect. Furthermore, the capacity of ALFQ to modulate MDM permissiveness to HIV-1 infection was predominantly mediated by large ALFQ liposomes. Our findings highlight a relationship between innate immune activation, proinflammatory milieu, and upregulation of anti-HIV proteins. Induction of these responses can switch the HIV-1 permissive MDM into a more refractory phenotype.

## Introduction

Macrophages are present in all tissues and are important in many immunological and physiological functions including phagocytosis, immune regulation, and host protection against pathogens. The protective role of macrophages is principally dependent on their ability to detect invading pathogens and to subsequently initiate an immune response. Detection is facilitated by an array of extracellular and intracellular pattern recognition receptors (PRRs), that when activated deliver signals through various adaptor molecules and kinases culminating in the production of various effector molecules including cytokines and chemokines^1,2^

Despite an array of extracellular and intracellular sensors for pathogens, macrophages are successfully hijacked by HIV-1^3,4^, and ultimately serve as a niche for virus replication. This paradoxical outcome in the macrophage/HIV-1 relationship is due to the ability of the virus to evade and/or actively subvert the cellular innate immune control pathways such as PRR signaling^5^**,** activation of transcription factors, induction of antiviral cytokines^6,7^, antiviral activity of miRNA, and antiviral restriction factors^8,9^. Tsang et al.,^3^ reported that macrophage innate sensors are incapable of sensing the presence of HIV-1 and therefore are unable to trigger innate cellular processes necessary for control of the viral infection. Additionally, it has been reported that the virus accessory protein, vpr, inhibits autophosphorylation of TANK-binding kinase 1 thus blocking the induction of type I and type III interferons which are necessary for the induction of interferon-stimulated genes to prevent infection^10^. Other studies showed that HIV-1 capsid interaction with specific cellular factors facilitates virus evasion of innate sensors without activation of innate immunity^4–11^.

The enhanced permissiveness of monocyte-derived macrophages (MDM) to HIV-1 contrasts with the resistance of circulating monocytes to HIV-1 infection. Recent studies have highlighted intracellular antiviral factors including tripartite motif (TRIM)5α, sterile alpha motif and histidine-aspartate domain-containing protein 1 (SAMHD1), tetherin, and the cytoplasmic apolipoprotein B mRNA-editing enzyme catalytic polypeptide-like (APOBEC) family as potent innate factors that are fundamental in the restriction of HIV-1 infection in myeloid cells^12,13^. APOBEC3A promotes macrophage polarization into a pro-inflammatory phenotype^14^, and maintains HIV-1 latency in infected cells^15^. While elevated in monocytes, expression of some of these antiviral factors is decreased in macrophages^16^. This may be governed by the environmental milieu in which the macrophages are undergoing differentiation and maturation and may be a likely contributing factor towards the permissiveness of MDM to HIV-1 infection. The question therefore arises whether the decreased expression of these antiviral factors can be reversed in differentiated macrophages to render them into a “monocyte-like phenotype” capable of restricting or limiting HIV-1 infection.

Immune modulators are principal activators of innate responses. Generally, immune modulators stimulate innate cells including monocytes and macrophages^17,18^ and activate a cascade of events culminating in the induction of a polarized immune response^17,19–21^. We utilized two adjuvants created in our laboratory, Army Liposome Formulation (ALF55) and ALFQ, to determine whether we could induce an innate response that would subsequently render primary human MDM refractory to HIV-1 infection. ALF55 contains saturated phospholipids, 55 mol % cholesterol and synthetic monophosphoryl lipid A (3D-PHAD®). ALFQ contains saturated phospholipids, 55 mol % cholesterol, synthetic monophosphoryl lipid A (3D-PHAD®) and a saponin, QS-21. This study is the first report highlighting comparative responses induced by ALF55 and ALFQ in MDM. ALF55 contains 3D-PHAD®, as an immunostimulant whereas ALFQ contains both 3D-PHAD® and QS-21. Monophosphoryl lipid A (MPLA) is a Toll-like receptor 4 (TLR4) agonist and stimulates NF-ĸB transcriptional activity for subsequent cytokine production^22^. QS-21 is a natural saponin fraction extracted from the bark of the South American tree *Quillaja saponaria Molina* and in combination with MPLA, was shown to activate the ACS-NLRP3 inflammasome and cause subsequent release of caspase-1 dependent proinflammatory cytokines IL-1β/IL-18^23^, which in turn act as potent inducers of an inflammatory milieu^20^. In this study, we show that pre-treatment of MDM with ALFQ enhanced inflammasome activation, induced a proinflammatory environment and upregulated cellular antiviral restriction factors, notably APOBEC3A. Together, these findings demonstrate a novel role for ALFQ in leveraging the innate restriction factors to render MDM derived in this environment less permissive to HIV-1 infection.

## Results

### ALFQ did not affect the viability of MDM

**Figure 1:**
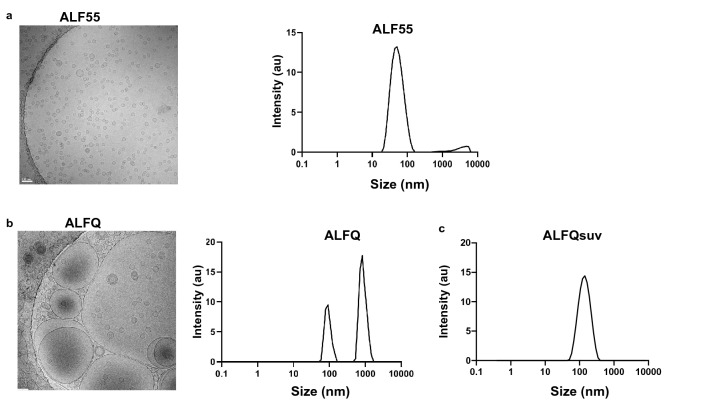
Visualization and sizing of ALF55 and ALFQ. ALF55, ALFQ, and ALFQsuv samples were visualized by cryo-electron microscopy (cryo-EM) and size distributions were measured by dynamic light scattering analysis (DLS). (**a**) Shows EM of small unilamellar vesicles in ALF55 (left) and their narrow size distribution (right). (**b**) Shows EM of both small and large vesicles in ALFQ (left) and a wider size distribution of the small and some large vesicles (right). However, DLS is not suitable to capture the range of the large size particles in ALFQ. **(c**) Shows the narrow size distribution of the vesicles in ALFQsuv.

**Figure 2:**
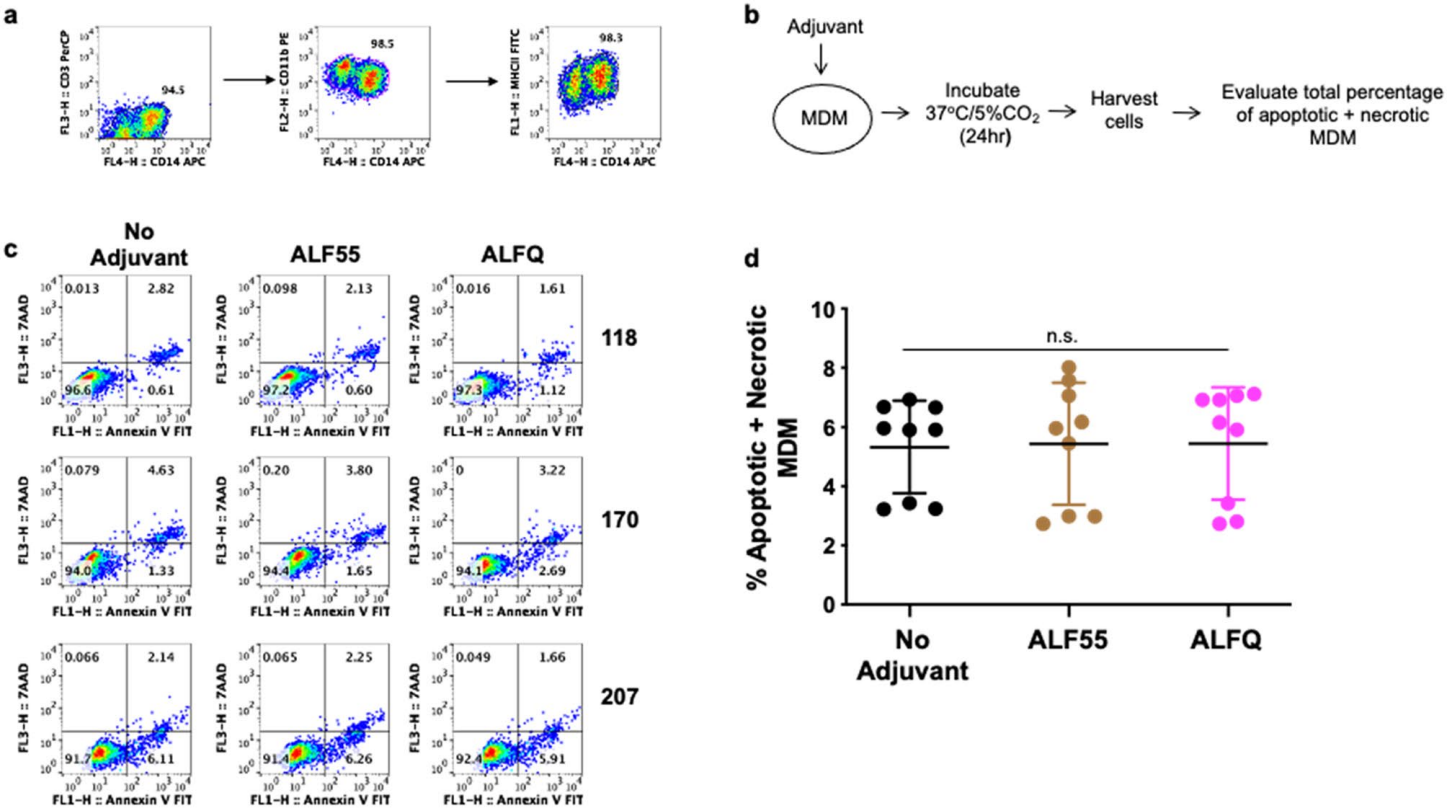
ALF55 and ALFQ do not induce apoptosis of MDM. Primary human monocytes were differentiated into MDM following in vitro culture with M-CSF media. (**a**) At day 4 post-culture, cells were harvested, stained and analyzed by flow cytometry. Plots show that the cells were CD14^+^CD11b^+^MHCII^+^ and did not contain CD3^+^T cells. (**b**) Scheme showing MDM treatment with adjuvants. (**c**) Day 4 MDM cultures were treated with ALF55 or ALFQ for 24 h. Untreated MDM (no adjuvant) cultures served as controls. Cultures were harvested, stained with a cocktail of anti-CD14, Annexin V FITC and 7AAD. The total percentage of apoptotic and necrotic CD14^+^ cells was analyzed by flow cytometry. Values in the bottom right and upper right quadrant(s) of the plots represent the percentage of apoptotic and necrotic MDM, respectively under each treatment. (**d**) Scatter dot plots represent the data from triplicate wells from the 3 donors (**c**) and show that the total percentage of apoptotic and necrotic MDM (mean ± s.d.) were similar in the untreated, ALF55-treated or ALFQ-treated MDM, as determined by One-way ANOVA. Data is representative of two independent experiments.

We first evaluated if ALF55 or ALFQ affected the viability of MDM. The characteristics of the liposome formulations are described in Table 1 and Fig. 1. ALF55 and ALFQsuv particles are small unilamellar nanovesicles (SUV) with a size range of 50–100 nm (Fig. 1a, c) whereas ALFQ is polydisperse and comprised both small and large unilamellar vesicles (Fig. 1b). The size of ALFQ vesicles ranged from 100 nm to several µm as shown in Fig. 1b, and from previously published data^24^. The MDM cultures were CD14^+^CD11b^+^MHCII^+^ and did not contain CD3^+^T cells (Fig. 2a). A schematic of the pre-treatment of MDM with the formulations is shown in Fig. 2b. The cells were harvested and analyzed for apoptosis and necrosis by Annexin V and 7AAD staining. Culture supernatants were analyzed for the presence of lactate dehydrogenase (LDH) as an indicator of pyroptosis. The percentages of apoptotic and necrotic MDM as measured by Annexin V and 7AAD staining was relatively low in the three donors and was comparable between the untreated (no adjuvant) and treated MDM cultures (Fig. 2c, d). Pre-treatment with ALF55 or ALFQ did not induce LDH production (data not shown).

**Table 1:** Composition of ALF55, ALFQ and ALFQsuv.

Adjuvant Formulation	Dispersity (Size)	DMPC/DMPG (mM)	Cholesterol (mol%)	3D-PHAD® (µg/ml)	QS-21 (µg/ml)	MPLA:Phospholipid
ALF55	SUV (0.1µ)	11.45	55%	200	0	1:88
ALFQ	Polydisperse (0.1µ up to 30µ)	11.45	55%	200	100	1:88
ALFQsuv	SUV (0.1µ)	11.45	55%	200	100	1:88

### ALFQ induced higher levels of proinflammatory cytokines and chemokines

**Figure 3:**
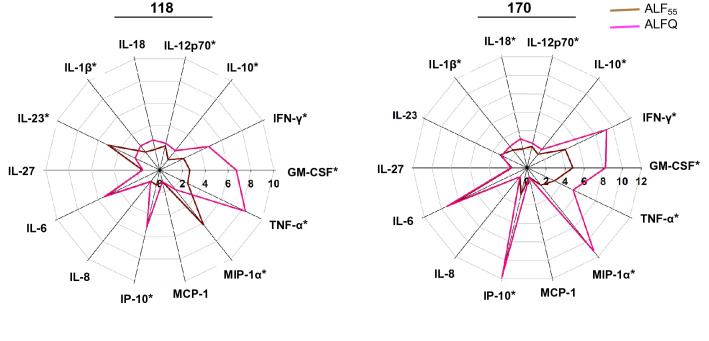
ALFQ induces a higher proinflammatory cytokine response. Primary human monocytes were differentiated into MDM following in vitro culture with M-CSF media. At day 4 post-culture, the cells were treated with ALF55 or ALFQ for 24 h. Untreated MDM cultures served as controls. Supernatants were harvested, clarified, and assayed for the presence of cytokines and chemokines using a custom-made U-Plex electrochemiluminescent immunoassay. Cytokine profiles were visualized as radar plots and show the increase (fold change) in cytokine levels induced by ALF55 (brown) or ALFQ (magenta) compared to unstimulated cultures. Data for each cytokine are averages of three independent experiments. Spokes represent the various cytokines tested; the origin of the plot is the baseline value of the respective cytokine in the unstimulated cultures. *p < 0.05, two-tailed Student t-test.

Having determined that ALF55 and ALFQ did not induce apoptosis of MDM (Fig. 2), we evaluated the profile and levels of cytokines and chemokines that were induced in the culture supernatants following exposure of MDM to ALF55 and ALFQ. The radar plots (Fig. 3) show the changes in the cytokine and chemokine levels induced by ALF55 or ALFQ in relation to the unstimulated MDM cultures.

Although the MDM from the two donors exhibited variability in the magnitude of their responses, culturing MDM with ALF55 and ALFQ induced significantly higher levels of all the cytokines and chemokines, except for MCP-1, compared to the media control. Comparative analyses between ALF55 and ALFQ showed induction of cytokine responses that were distinct in both magnitude and profile. MDM cultured with ALFQ generally secreted higher levels of proinflammatory cytokines and chemokines than MDM cultured with ALF55 as reflected by the significantly higher levels of IL-12p70, IFNγ, GM-CSF, IL-1β, IL-18, TNF-α, IP-10 and MIP-1α (Fig. 3). ALF55 or ALFQ did not induce IFNα in any of the donors. These results indicate that MDM respond to different environmental stimuli during their differentiation process, and this determines the profile and magnitude of soluble mediators that are secreted in the supernatant.

### ALFQ upregulated expression of MHC-II and CD86

**Figure 4:**
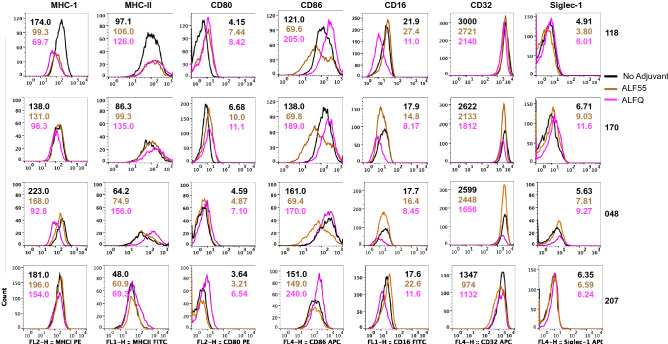
MDM exposed to ALFQ upregulate MHC-II and co-stimulatory molecules. Primary human monocytes were differentiated into MDM following in vitro culture with M-CSF media. At day 4 post-culture, the cells were treated with ALF55 or ALFQ for 24 h. Untreated MDM (no adjuvant) cultures served as controls. Cells were harvested, stained, and analyzed for the expression of MHC-I, MHC-II, CD80, CD86, CD16, CD32 and Siglec-1. Histogram overlays show the expression of the indicated cell receptor on the gated CD14^+^MDM that were not treated with any adjuvant (black line) or were treated with ALF55 (brown line) or ALFQ (magenta line). Values shown inside the histograms are the mean fluorescent intensity (MFI) of the specific stain. Data are representative of triplicate wells of two independent experiments for four donors.

**Figure 5:**
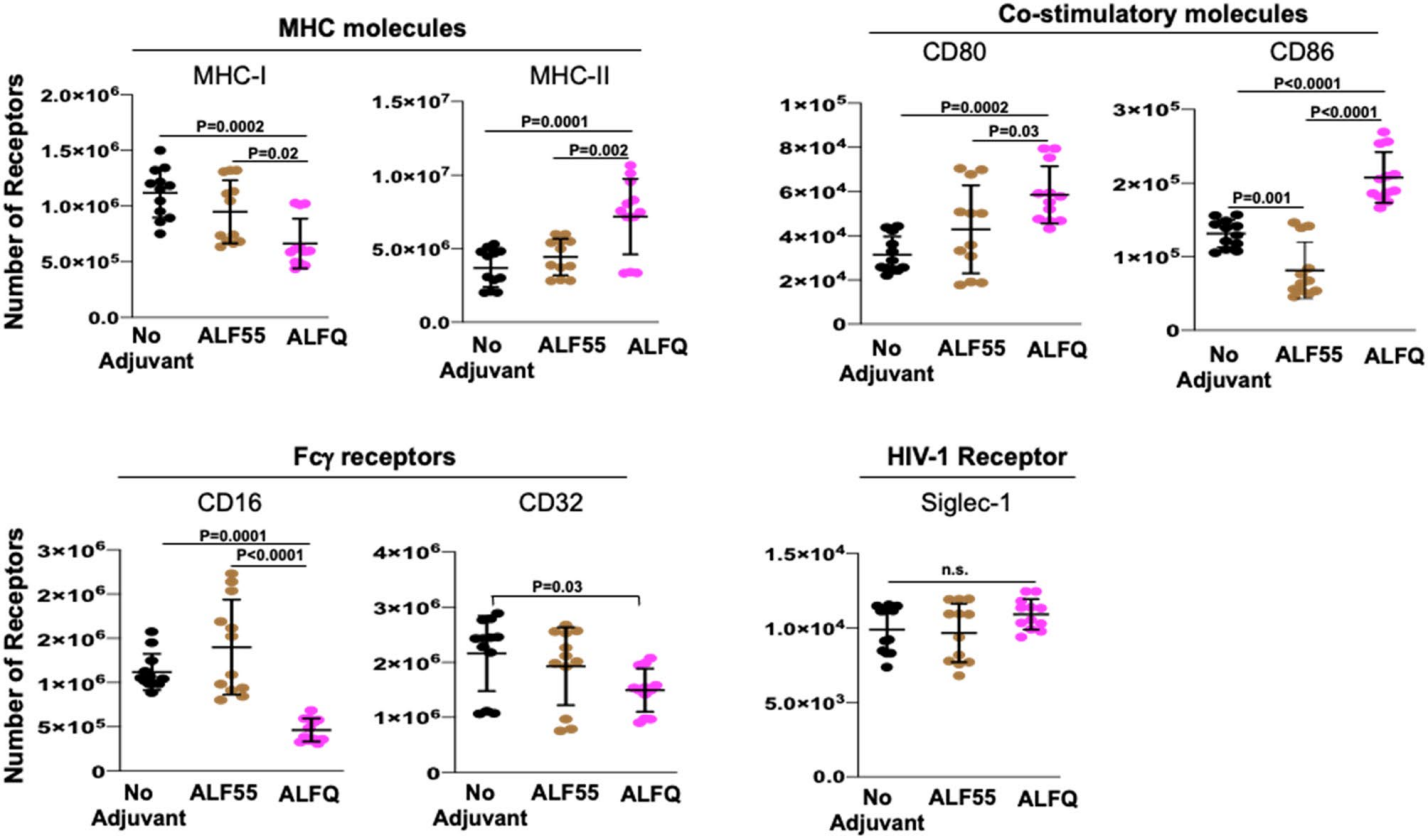
Receptor numbers on MDM are differentially affected by ALFQ. Primary human monocytes were differentiated into MDM following culture with M-CSF media. At day 4 post-culture, the cells were treated with ALF55 or ALFQ for 24 h. Untreated MDM (no adjuvant) cultures served as controls. Cells were harvested, stained, and the number of MHC-I, MHC-II, CD80, CD86, CD16, CD32 and Siglec-1 receptors was quantified by flow cytometry. Scatter dot plots show the number of the indicated cell receptors on the gated CD14^+^MDM that were not treated with any adjuvant (black circles) or were treated with ALF55 (brown circles) or ALFQ (magenta circles) for triplicate wells for the four donors shown in Fig. 4. Data are representative of two independent experiments. *P* values indicate statistical differences by One-way ANOVA.

We characterized the effect of ALF55 and ALFQ on the expression of antigen presentation receptors (MHC-I, MHC-II), co-stimulatory receptors (CD80, CD86), phagocytosis-associated receptors (CD16, CD32) and HIV-capture associated receptor sialic acid-binding immunoglobulin-like lectin-1 (Siglec-1) on MDM. We previously demonstrated that Siglec-1 on MDM is an important attachment receptor for HIV-1 and has a profound influence on HIV-1 infectivity^25^. The surface expression of these receptors was evaluated by assessment of the mean fluorescent intensity (MFI) (Fig. 4) and the quantitation (Fig. 5) of the cellular receptors 24 h post-treatment with the adjuvants. ALF55 and ALFQ differentially influenced MHC receptors, co-stimulatory molecules and Fc-gamma receptors. ALFQ significantly increased the expression of MHC-II, CD80, and CD86 and correspondingly decreased the expression of CD16 when compared to ALF55 or the unstimulated (no adjuvant) controls (Fig. 5). Interestingly, ALF55 significantly decreased the expression of CD86 when compared to the unstimulated controls (Fig. 5). Although the number of CD32 receptors was comparable between MDM exposed to ALFQ or ALF55, they were significantly lower in the ALFQ-exposed MDM when compared to the unstimulated controls. The number of MHC-I receptors was comparable between ALF55-exposed MDM and the unstimulated controls. In contrast, MHC-I expression was significantly decreased in the ALFQ-exposed MDM when compared to ALF55-exposed or the unstimulated (no adjuvant) controls (Fig. 5). These results suggest that ALFQ could enhance the capacity of MDM to present antigen through the MHC-II antigen presentation pathway with a concomitant modulation in receptor-mediated phagocytosis. The observed effects may be a direct consequence of the immunomodulator(s) or an indirect result of the cytokines secreted in response to immunomodulators that influences the immune environment in which the cells are undergoing differentiation.

### Pre-treatment with ALFQ modulates permissiveness of MDM to HIV-1 infection

**Figure 6:**
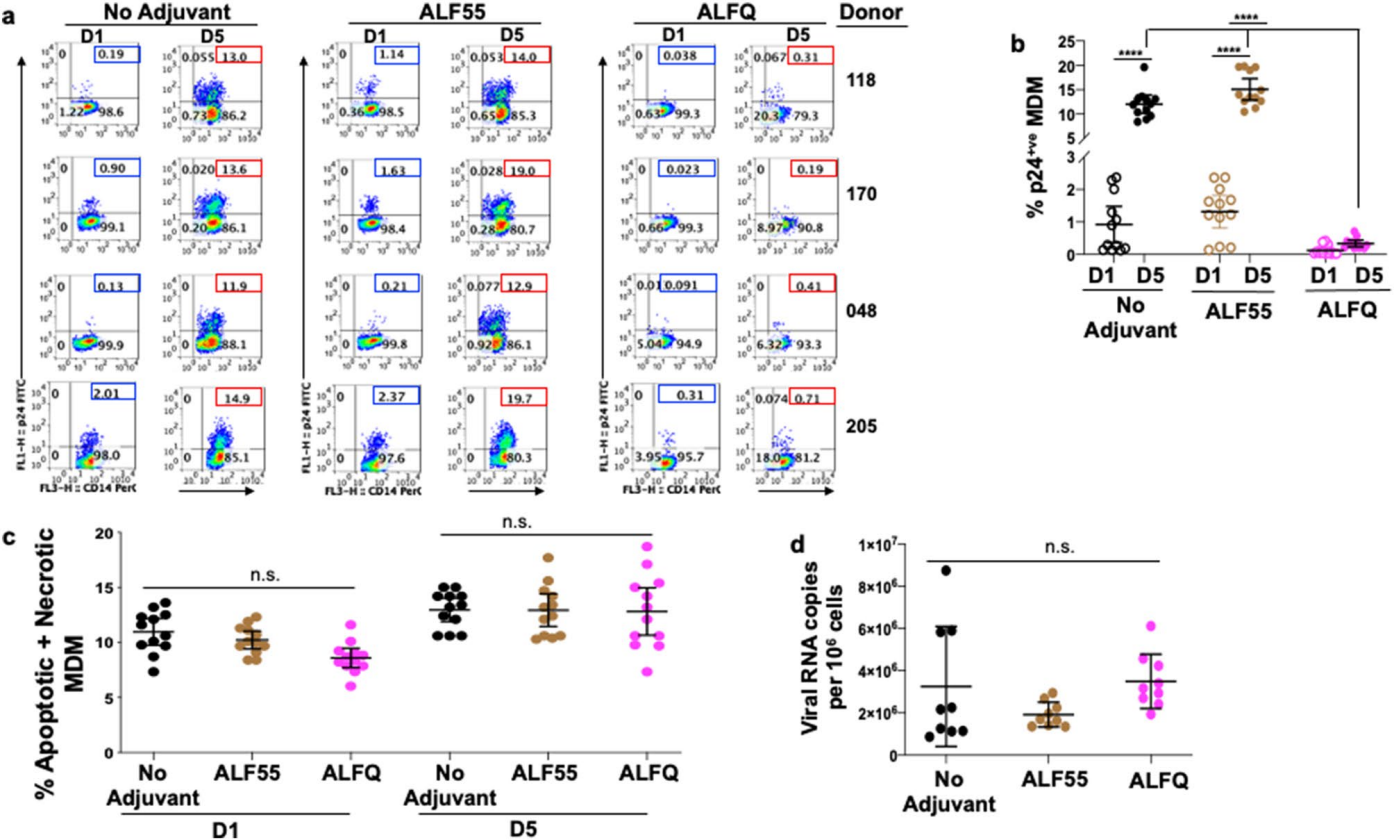
HIV-1 infection is restricted in ALFQ-treated MDM. Primary human monocytes were differentiated into MDM following in vitro culture with M-CSF media. At day 4 post-culture, the cells were pre-treated with ALF55 or ALFQ for 24 h. Untreated MDM (no adjuvant) cultures served as controls. Cells were washed and infected with US-1 (4 ng p24). Cultures were harvested at day 1 and day 5 post-infection (p.i) and analyzed for the presence of intracellular p24 by flow cytometry. (**a**) Values (blue boxes, day 1 p.i; red boxes, day 5 p.i) in the upper right quadrant(s) of the plots represent the percentage of HIV-1-infected MDM under each treatment. (**b**) Scatter dot plots (open circles, day 1 p.i; filled circles, day 5 p.i) represent the data from triplicate wells of 4 donors and show the percentage of HIV-1-infected MDM (mean ± s.d.) in untreated (no adjuvant) MDM (black circles), MDM treated with ALF55 (brown circles) or with ALFQ (magenta circles). Statistical difference between the treatments was determined by One-way ANOVA. Statistically significant differences between % of infected cells at day 1 and day 5 p.i for each of the treatments as well as differences in % of infected cells between ALF55- and ALFQ-treated MDM at day 5 p.i. are shown. ****P < 0.0001. (**c**) Scatter dot plots show the percentage (mean ± s.d.) of apoptotic and necrotic MDM in the untreated cultures (black circles), cultures treated with ALF55 (brown circles) or ALFQ (magenta circles) at day 1 and day 5 post-infection of triplicate wells of four donors. Two independent experiments were conducted. The percentage (mean ± s.d.) of apoptotic and necrotic MDM was compared between the groups by One-way ANOVA and were not statistically different (n.s.) at each time-point. (**d**) Scatter dot plots represent the amount of captured virus and show the amount of HIV-1 RNA per 10^6^ MDM (mean ± s.d.) in untreated (no adjuvant) MDM (black circles), MDM treated with ALF55 (brown circles) or with ALFQ (magenta circles). of triplicate wells of three donors. The groups were compared by One-way ANOVA and were not statistically different.

We previously demonstrated that MDM comprised two populations: a non-adherent (CD14^+^CD4^+^Siglec-1^hi^) population refractory to HIV-1 infection and an adherent (CD14^+^CD4^-^Siglec-1^ l^°) population which was highly permissive to HIV-1 infection^26^. Therefore, we assessed the influence of ALF55 and ALFQ on the permissiveness of adherent MDM to HIV-1 infection. MDM were either untreated (no adjuvant) and served as infection control or pre-treated with ALF55 or ALFQ for 24 h. Non-adherent MDM were removed, and the remaining adherent MDM were subsequently infected with US-1 as previously described^26^. MDM were harvested on day(s) 1 and 5 post-infection and analyzed for the presence of intracellular p24 by flow cytometry (Fig. 6a, b). When compared to the infection controls, pre-treatment with ALF55 or ALFQ did not modulate the permissiveness of the MDM to HIV-1 infection at day 1 post-infection. In contrast, at day 5 post-infection, pre-treatment with ALFQ significantly decreased the percentage of HIV-1-infected MDM in all donors when compared to either the untreated MDM (no adjuvant) or MDM that were pre-treated with ALF55 (Fig. 6a, b). Furthermore, whereas the percentage of HIV-1-infected MDM was not statistically different between day 1 and day 5 post-infection in the ALFQ-treated MDM, they were significantly increased between day 1 and day 5 post-infection in the untreated and ALF55 pre-treated MDM. These results indicate that exposure to ALFQ render adherent MDM significantly less permissive to HIV-1 infection.

One probable reason for the lower number of infected cells in the ALFQ-treated cultures may be because infected MDM from the ALFQ-treated cultures became apoptotic instead of being productively infected. Although our data (Fig. 2) showed that ALFQ by itself did not induce apoptosis or pyroptosis, it is possible that ALFQ-treated cultures may be more susceptible to apoptosis when infected with HIV-1. However, as shown in Fig. 6c, the percentages of apoptotic and necrotic MDM were comparable between the untreated (no adjuvant) and adjuvant-treated MDM at each time-point and ranged between 7.3 and 13.6% in the untreated cultures, 8.4–11.9% for ALF55 and 6.1–9.2% for ALFQ at day 1 post infection., and 10.6–14.2% in the untreated cultures, 10.6–17.7% for ALF55 and 10.6–18.7% for ALFQ at day 5 post infection. (Fig. 6c), suggesting that apoptosis and or necrosis of HIV-1 infected cells was not the cause of reduced infection in ALFQ treated cultures.

Another potential reason for the lower number of infected cells in the ALFQ-treated cultures may be because ALFQ modulated the number of viruses that was captured by the ALFQ-treated cultures during the initial HIV-1 exposure. Although our data (Fig. 5) showed that ALFQ did not modulate expression of Siglec-1, an important HIV-1 attachment receptor, we determined whether HIV-1 capture and entry was decreased in the ALFQ-treated MDM. Cells were lysed 1 h post-infection of the MDM and virus capture and entry was determined by the presence of RNA gag using qRT-PCR^27^. The number of HIV-1 RNA copies per 10^6^ MDM was comparable in the untreated and adjuvant-treated MDM (Fig. 6d) suggesting that pre-treatment with ALFQ did not modulate attachment and entry of HIV-1 in MDM, but influenced the HIV-1 replication capacity.

**Figure 7:**
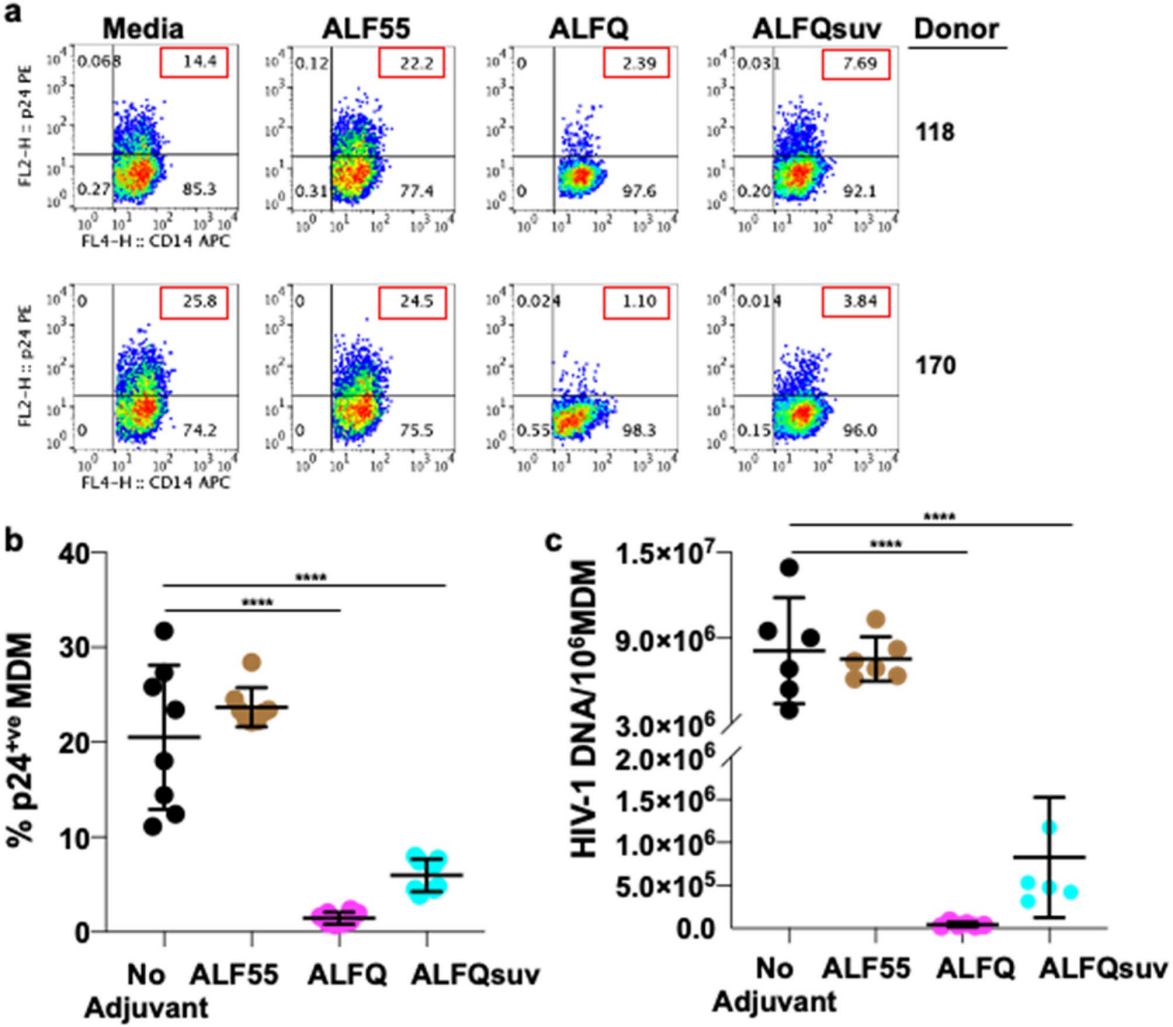
Large liposomes principally mediate restriction of HIV-1 infection in ALFQ-treated MDM. Primary human monocytes were differentiated into MDM following in vitro culture with M-CSF media. At day 4 post-culture, the cells were treated with ALF55, ALFQ, or ALFQsuv for 24 h. Untreated MDM (no adjuvant) cultures served as controls. Cells were washed and infected with US-1 (4 ng p24). Cultures were harvested at day 5 post-infection (p.i) and analyzed for the presence of intracellular p24 by flow cytometry and HIV-1 DNA content by qPCR. (**a**) Values (red boxes) in the upper right quadrant(s) of the flow plots represent the percentage of HIV-1-infected MDM under each treatment. (**b**) Scatter dot plots represent the data from quadruplicate wells of two donors (**a**) and show the percentage of HIV-1-infected MDM (mean ± s.d.) in untreated MDM (black circles), MDM treated with ALF55 (brown circles), ALFQ (magenta circles) or with ALFQsuv (cyan circles). (**c**) Scatter dot plots show the amount of HIV-1 per 10^6^ HIV-1-infected MDM (mean ± s.d.) in untreated MDM (black circles), MDM treated with ALF55 (brown circles), ALFQ (magenta circles) or with ALFQsuv (cyan circles) from triplicate wells of the two donors. Statistical differences between the groups were determined by One-way ANOVA. ****P < 0.0001.

As shown in Fig. 1b, ALFQ is polydisperse and comprise both small and large unilamellar vesicles. In order to determine whether the decreased permissiveness to HIV-1 infection following exposure of MDM to ALFQ was influenced by liposome size, ALFQ was microfluidized to generate small ALFQ liposomes (ALFQsuv). MDM were either untreated (no adjuvant) and served as infection control or pre-treated with ALF55_,_ ALFQ or ALFQsuv for 24 h. Non-adherent MDM were removed, and the remaining adherent MDM were subsequently infected with US-1 for 5 days. Notably, pre-treatment with ALFQ or ALFQsuv significantly decreased the percentage of HIV-1-infected MDM at day 5 post-infection when compared to the untreated or ALF55 pre-treated MDM (Fig. 7a, b). When compared to the untreated MDM, pre-treatment with ALFQ decreased HIV-1 infectivity by sixfold and 23.4-fold, respectively for donors 118 and 170. However, in the presence of ALFQsuv, the decrease in infectivity was only 1.9-fold and 6.7-fold respectively, for the two donors. Furthermore, quantitative analyses of the HIV-1 DNA in the MDM at day 5 post-infection showed that pre-treatment with ALFQ resulted in lower levels of viral DNA when compared to MDM that were pre-treated with ALFQsuv or ALF55 (Fig. 7c). This suggests either that the capacity of ALFQ to modulate MDM permissiveness to HIV-1 infection was principally mediated by the large ALFQ liposomes per se, or by the greater number of QS-21 molecules embedded on the outer surfaces of the large ALFQ liposomes when compared to ALFQsuv that have a large fraction of QS-21 molecules embedded on the inner leaflets of the outer lamellae. The presence of QS-21 on the outer and inner lamellae of the vesicles is a topic of further research.

### ALFQ up-regulates APOBEC3A gene expression and protein synthesis in adherent MDM

**Figure 8:**
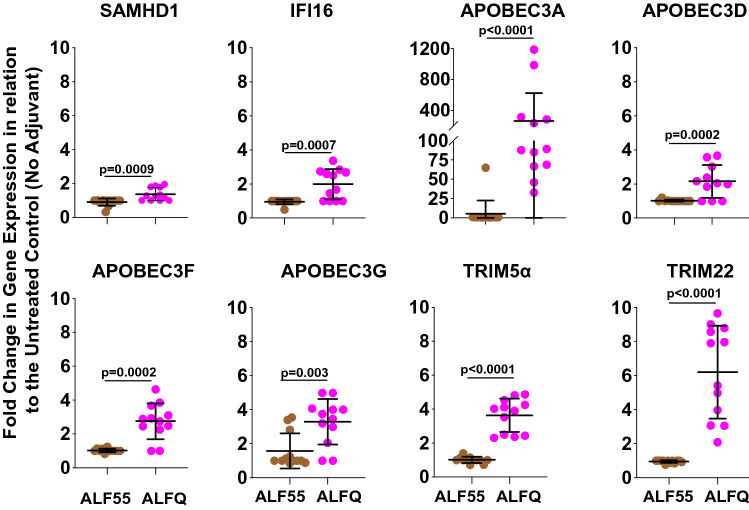
Expression of anti-viral genes is upregulated in ALFQ-treated MDM. Primary human monocytes were differentiated into MDM following in vitro culture with M-CSF media. At day 4 post-culture, the cells were treated with ALF55 or ALFQ for 24 h. Untreated MDM cultures served as controls. Cells were harvested, lysed and the gene expression levels of the cellular restriction factors were analyzed by qRT-PCR. The dot plots show fold-change in the expression levels of HIV-1 restriction genes for MDM that were pre-treated with ALF55 (brown circles) or ALFQ (magenta circles) in relation to the untreated (no adjuvant) MDM from triplicates wells of 4 donors. Statistical differences between the two groups were determined by two-tailed Mann–Whitney test.

Antiviral cellular restriction factors modulate HIV-1 infection. To gain more insight into the possible reasons for the restricted permissiveness to HIV-1 infection in the adherent MDM from the ALFQ-treated cultures, the expression of several antiviral cellular restriction factors was compared in adherent MDM pre-treated with ALF55, ALFQ or without any adjuvant (Fig. 8). Based on our previous study where we observed a differential expression of HIV-1 restriction factors in MDM subsets using RNA-Seq^26^, we evaluated the gene expression of SAMHD1, IFI16, APOBEC family, TRIM5α and TRIM22 in lysates of the adherent MDM by qRT-PCR. The data are plotted as fold change in gene expression in adherent MDM pre-treated with ALF55 or ALFQ in relation to adherent MDM without any adjuvant treatment. Pre-treatment with ALF55 did not induce any increase in gene expression of the restriction factors tested. In contrast, ALFQ significantly upregulated the gene expression of SAMHD1, IFI16, APOBEC3 families, TRIM5α, and TRIM22 to varying degrees. The most noticeable effect of ALFQ was observed in approximately 30- to 1100-fold higher expression of APOBEC3A (Fig. 8).

**Figure 9:**
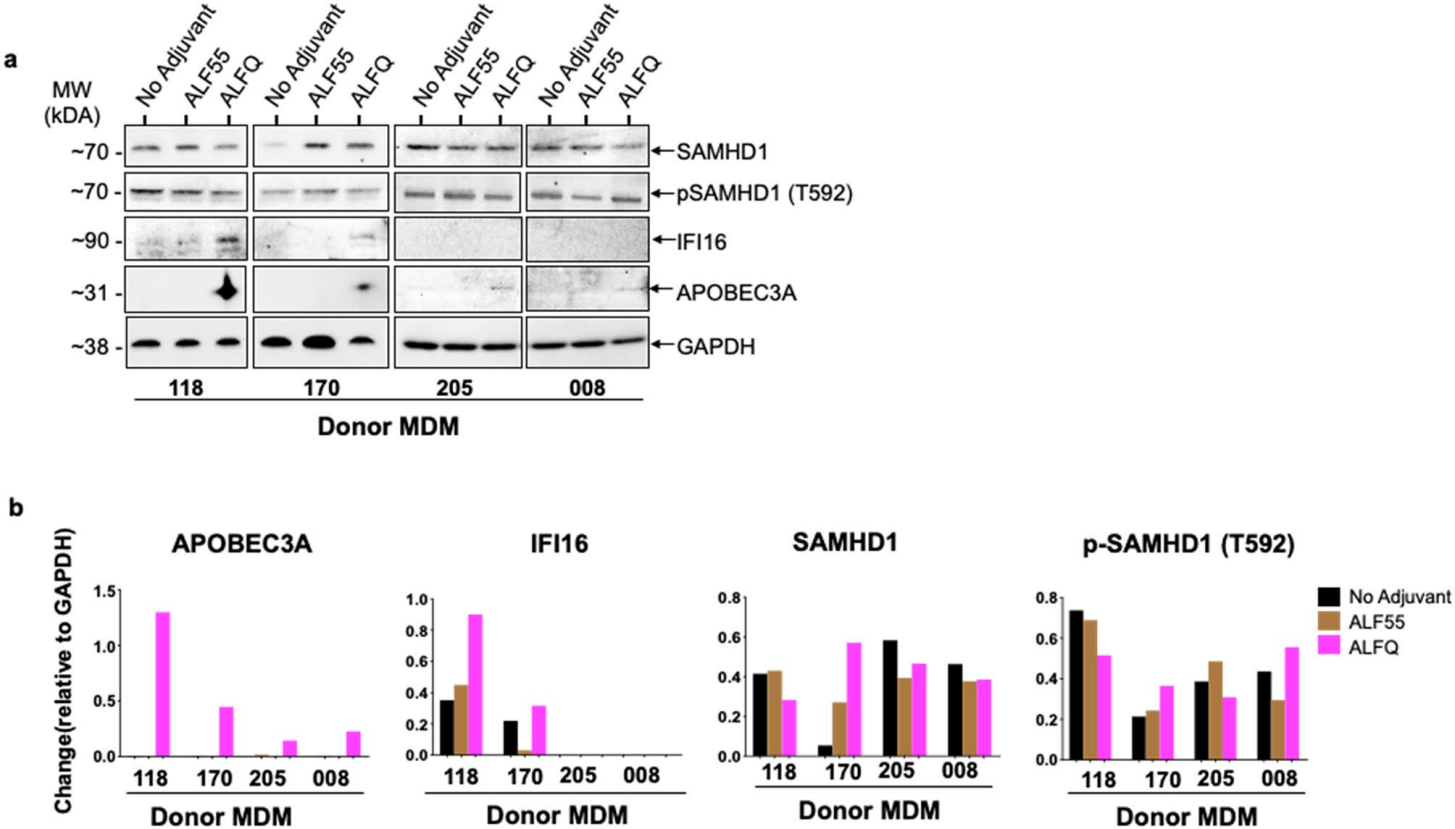
Protein synthesis of APOBEC3A and IFI16 is induced in ALFQ-treated MDM. Primary human monocytes were differentiated into MDM following in vitro culture with M-CSF media. At day 4 post-culture, the cells were treated with ALF55 or ALFQ for 24 h. Untreated (no adjuvant) MDM cultures served as controls. Cells were harvested, lysed, and analyzed by SDS-PAGE followed by Western blotting using antibodies against SAMHD1, phospho-SAMHD1, IFI16 and APOBEC3A. (**a**) Cropped images of the blots shows the presence of IFI16 protein and APOBEC3A protein only in lysates from ALFQ-treated MDM. (**b**) Densitometry analysis was performed on protein bands (APOBEC3A, IFI16, SAMHD1, and phospho-SAMHD1), and normalized to its corresponding GAPDH band. The original blots are presented in Supplementary Figs. 1–5.

In order to determine if the increased gene expression in adherent MDM from the ALFQ-treated MDM cultures was also accompanied by a concomitant increase in protein synthesis, lysates of adherent MDM from ALF55-treated, ALFQ-treated, and adjuvant-untreated cultures were subjected to SDS-PAGE followed by Western blotting with antibodies against SAMHD1, IFI16, APOBEC3A, and GAPDH. APOBEC3A protein was only detected in the lysates of the ALFQ-treated cultures. The APOBEC3A band was detected in all 4 donors. However, the APOBEC3A band was more prominent in the lysates from donors #170 and #118 when compared to donors #205 and #008. We believe that the observed differences could be due to the use of anti-APOBEC3A polyclonal antibodies from two different sources. The use of a different source for anti-APOBEC3A antibody was due to supply chain difficulties. IFI16 protein was detected under all conditions with donor #118 although the level of protein expression was higher in the lysates of the ALFQ-treated cultures. Interestingly, in donor #170, IFI16 protein was detected primarily in ALFQ- and not in ALF55-treated cultures. The difference in IFI16 levels between donors #118 and #170 suggests that ALF55 may have a differential effect on IFI16 expression in different donors. IFI16 protein was not detected with donors #205 and #008. SAMHD1 protein was detected under all conditions in all the donors and further analyses with an antibody against phosphorylated-Thr592-SAMHD1 (p-SAMHD1) revealed phosphorylation on Thr592 residue in all four donors under all conditions. (Fig. 9a and S1–S5).

Quantitation of the Western blot bands by densitometry analysis (Fig. 9b), and normalization of each band against GAPDH, confirmed the presence of APOBEC3A levels exclusively in ALFQ-treated cultures and an increase in IFI16 levels upon treatment with ALFQ. Quantitation of SAMHD1 bands showed a differential effect with ALFQ treatment which varied from donor-to-donor. Whereas ALFQ decreased the level of SAMHD1 in donors #118, #205 and #008 in relation to the untreated (no adjuvant) cultures, the level of SAMHD1 was increased in donor #170. Treatment with ALFQ increased the level of phosphorylated SAMHD1 (p-SAMHD1) in donors #170 and #008 in relation to both the untreated and ALF55-treated cultures. The level of p-SAMHD1 was decreased in ALFQ-treated cultures of donors #118 and #205.

**Figure 10:**
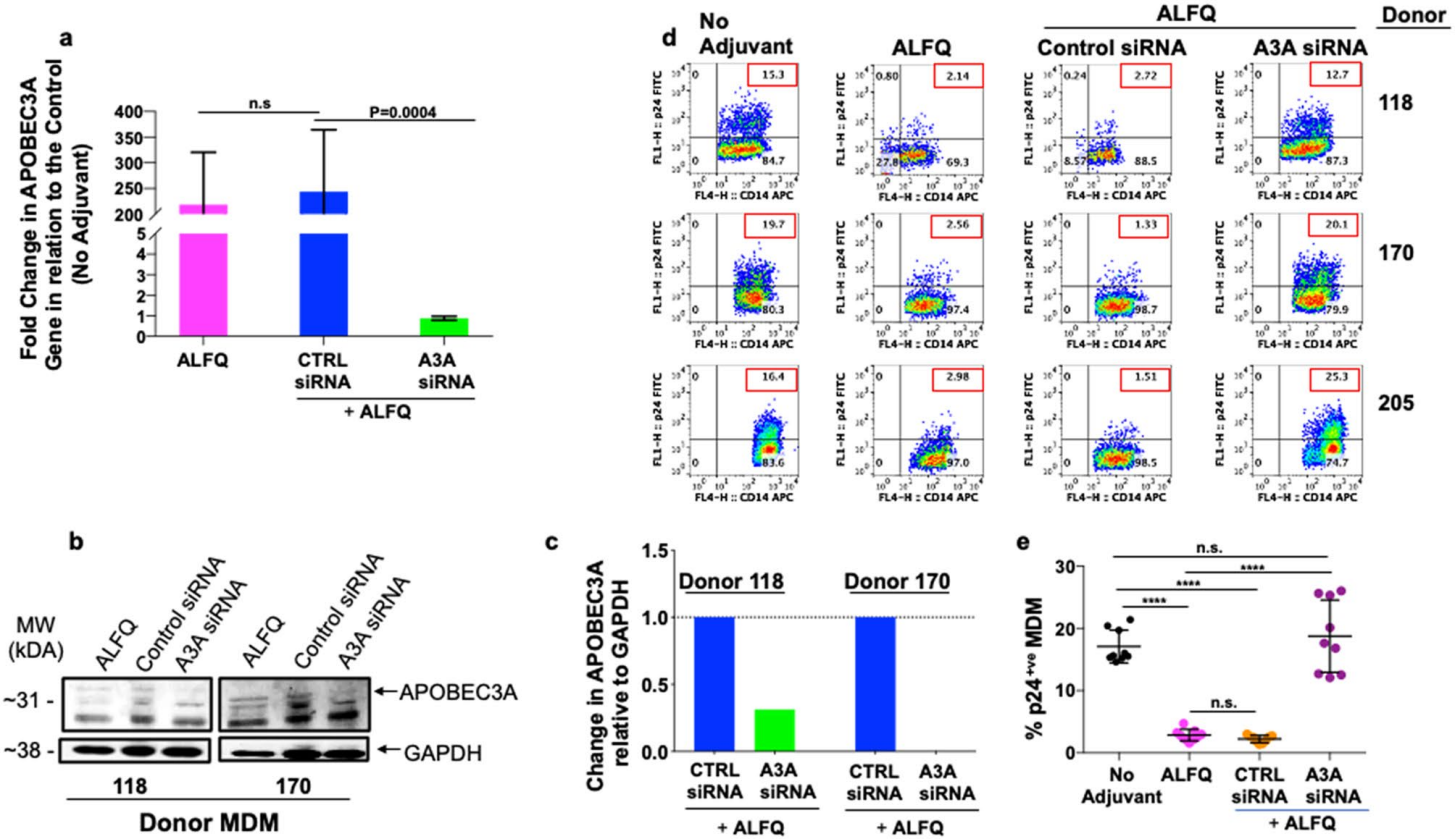
APOBEC3A is involved in the modulation of HIV-1 infection in ALFQ-treated MDM. Primary human monocytes were differentiated into MDM following in vitro culture with M-CSF media. At day 3, cultures were transfected with either APOBEC3A siRNA (A3A siRNA) or with scrambled siRNA (Control siRNA) for 24 h. At day 4 post-culture, the transfected cultures were treated with ALFQ for 24 h. MDM cultures that were neither transfected nor treated with ALFQ, or not transfected but treated with ALFQ at day 4 post-culture served as controls. (**a**) Cells from 3 donors were harvested, lysed and the gene expression levels of APOBEC3A were analyzed by RT-PCR. Bar graph shows fold-change (mean ± SEM) in the expression levels of APOBEC3A gene in relation to the untreated (no adjuvant) MDM controls. Data was analyzed using One-way ANOVA. (**b**) Cropped Western blotting (the upper band at 31kDA represents APOBEC3A) and (**c**) densitometry analyses show a decrease in APOBEC3A protein in the lysates of A3A siRNA cultures when compared to lysates of Control siRNA cultures. The original blot is presented in Supplementary Figs. 1–5. (**d**) Plots shows the percentage of intracellular p24 by flow cytometry at day 5 post-infection with HIV-1 in 3 donors (described in **a**) either not transfected or transfected with siRNA before treatment with ALFQ. Values in the upper right quadrant(s) of the plots represent the percentage of HIV-1-infected MDM under each treatment. (**e**) Scatter dot plot represent the data from (**d**) for triplicate wells from 3 donors and show the percentage of HIV-1-infected MDM (mean ± s.d.) in untreated MDM (black circles), MDM treated with ALFQ (magenta circles), A3AsiRNA-transfected-ALFQ-treated MDM (orange circles), and scrambled siRNA-transfected-ALFQ-treated MDM (purple circles) of triplicate wells from the 3 donors. ****P < 0.0001, One-way ANOVA.

APOBEC3A restrict HIV-1 infection in myeloid cells. Silencing of APOBEC3A gene in monocytes render them permissive to HIV-1 infection whereas, induction of APOBEC3A in MDM render them more resistant to HIV-1 infection^16^. To further evaluate the immunomodulatory role of APOBEC3A in restriction of HIV-1 infection, MDM were untreated or treated with control siRNA or APOBEC3A siRNA (A3A siRNA) 24 h prior to ALFQ treatment (Fig. 10 and S6). The MDM cultures were subsequently infected with HIV-1. Silencing was done using a mix of two human APOBEC3A siRNAs. There was no significant difference in APOBEC3A gene expression between ALFQ treated and control siRNA treated MDM. However, treatment with A3A siRNA resulted in a significant decrease in APOBEC3A gene expression (Fig. 10a), a concomitant decrease in APOBEC3A protein synthesis was also observed by Western blotting (Fig. 10b and S6) and densitometry (Fig. 10c) with an increase in HIV-1 infectivity (Fig. 10d, e). HIV-1 infection of MDM treated with A3AsiRNA resulted in a significant loss in the restriction of HIV-1 infectivity in all 3 donors tested (Fig. 10d), resulting in a similar percentage of p24 positive MDMs as untreated MDM, whereas control siRNA-treated MDM showed similar results (restriction of infection) as ALFQ-treated MDM (Fig. 10d, e). The data from Figs. 9 and 10, demonstrate that the induction of APOBEC3A by ALFQ restricts HIV-1 infection in ALFQ-treated MDM.

## Discussion

Our data highlight interesting and novel differences on the effects of Army Liposome Formulations on MDM, particularly the ALFQ formulation which contains QS-21. Exposure of MDM to ALFQ which contains two immunostimulants synthetic monophosphoryl lipid A (3D-PHAD®) and QS-21, modulated permissiveness of the MDM to HIV-1 infection. This was concomitant with increased expression of various innate anti-HIV restriction genes and proteins particularly APOBEC3A, inflammasome activation, increased production of proinflammatory cytokines and chemokines, and increased surface expression of MHC-II and the co-stimulatory molecules CD80 and CD86. M1 macrophages are associated with increased CD86 expression^14,28^, and polarization towards this phenotype is largely dependent on environmental cues^29,30^. In contrast, exposure of MDM to ALF55 which contains only one immunostimulant, 3D-PHAD®, induced lower levels of proinflammatory mediators, had minimal or no effect on anti-HIV restriction genes and did not make MDM less permissive to HIV-1 infection. Our previous studies using RNA-seq^26^ had demonstrated reduced expression of innate restriction factors in adherent MDM, which contributed to increased permissiveness to HIV-1. In the present study we evaluated if ALFQ would positively affect the expression of the innate restriction factors, which in turn would modulate permissiveness to HIV-1 infection. Our results demonstrate that exposure of MDM to ALFQ, but not ALF55 induced increased gene expression of APOBEC3A and IFI16 as determined by qRT-PCR with a concomitant increase in protein levels. Furthermore, exposure of MDM to ALFQ also elicited the secretion of proinflammatory effector mediators creating an inflammatory environment, thus polarizing the MDM into an inflammatory phenotype. The cumulative effects of these induced responses may be pivotal in rendering MDM significantly less permissive to HIV-1 infection.

ALFQ and ALF55 are unilamellar liposomes with identical phospholipid and cholesterol concentrations. However, ALF55 comprises SUVs (0.1 µ), while ALFQ is polydisperse and contains both small and large liposomes. The size of the small liposomes ranges from 50 to 100 nm as opposed to the large liposomes which can be ≥ 3000 nm in diameter^31^_′_^32^. The outer surface of the ALFQ particle has a “sugar lawn” consisting of ten different sugars (two on MPLA, and eight on QS-21) which could serve as ligands for binding to cell receptors. In this regard, the larger ALFQ liposomes may have increased visibility compared to small ALFQ, which could influence the outcome of the MDM responses. Our study showed that small ALFQ liposomes (ALFQsuv) had a significantly reduced capacity to modulate permissiveness of MDM to HIV-1 infection when compared to ALFQ which indicates that either the liposome size, or the placement of QS-21 on the outer leaflet of the outer lamella (ALFQ), or on both the outer and inner leaflets of the outer lamella (ALFsuv), greatly influenced the effects of ALFQ on MDM. However, ALF55 which are also SUVs did not exhibit the same effects on MDM as ALFQsuv in modulating MDM permissiveness to HIV-1 infection. Therefore, size may not be the only critical factor. The major chemical difference between the 3 liposomes is the presence of QS-21 in ALFQ and ALFQsuv but not in ALF55. Free QS-21 is highly toxic, however when QS-21 is present in liposomes, it irreversibly binds to cholesterol in the liposomes and loses its toxic effect but maintains its adjuvanticity^33^. The precise role of QS-21 in ALFQ is poorly characterized. Free QS-21 in combination with MPLA (presumably micellar) has been shown to activate the ACS-NLRP3 inflammasome and cause the subsequent release of caspase-1 dependent proinflammatory cytokines IL-1β/IL-18 in in vitro studies with mouse APCs^23^. We observed similar results when both QS-21 and MPLA were formulated in liposomes as ALFQ.

IL-1β and IL-18 are the signature cytokines of inflammasome activation. Although both cytokines were induced by ALF55 and ALFQ, the levels were significantly higher in response to ALFQ thus indicating that the QS21-containing adjuvants are superior inflammasome activators. Although inflammasome activation is associated with pyroptotic cell death, neither ALF55 nor ALFQ elicited increased LDH production in the MDM cultures. Indeed, the amount of LDH production in the MDM cultures exposed to ALF55 or ALFQ was similar to the levels detected in the control cultures that were not exposed to any of the adjuvants. Our observation that MDM may secrete IL-1β or IL-18 and not undergo pyroptosis is in line with recent reports that highlight the capability of myeloid cells to secrete IL-1β in a GSDMD-independent manner^34,35^, and other studies in which viable monocytes, macrophages, and neutrophils have been shown to secrete IL-1β and not undergo pyroptosis^34,36^.

The difference in permissiveness to HIV-1 infection between MDM that were exposed to ALFQ compared to those that were exposed to ALF55 was independent of cellular expression of Siglec-1 or the capacity to capture HIV-1 virions. However, quantitative analyses of the HIV-1 DNA from ALFQ pre-treated MDM showed significantly lower levels of viral DNA when compared to MDM that were pre-treated with ALF55 (Fig. 7c), thereby indicating that although ALFQ did not inhibit the capture and entry of HIV-1 (Fig. 6c), ALFQ inhibited the replication phase.

Earlier reports have shown that monocytes are largely refractory to HIV-1 infection^37^, due to the presence of innate anti-HIV-1 genes and proteins including APOBEC3^16^ and SAMHD1^12^ proteins. The differentiation process into MDM is associated with down-regulation of some of these innate anti-HIV-1 genes rendering the MDM highly permissive to HIV-1 infection^16,38^. In addition, although cells may express SAMHD1, the anti-HIV-1 activity of SAMHD1 is lost when it is phosphorylated at position T592. This results in a loss in the ability of the cell to restrict HIV-1 infection^39,40^. Our results show that there was a donor-to-donor variation in the levels of SAMHD1 as well as in p-SAMHD1 in ALFQ treated MDM. The level of p-SAMHD1 was decreased in some donors, while in others it was slightly increased compared to untreated MDMs. Thus, it is possible that more than one restriction factor is involved in modulating HIV-1 infection.

Our data further indicates a differential effect on innate anti-HIV-1 genes that are associated with modulation of HIV-1 infection in MDM. Innate sensors including IFI16 are necessary for cells such as macrophages to sense the intracellular presence of HIV-1 and subsequently trigger innate cellular processes necessary for control of the viral infection^3^. The innate cellular HIV-1 restriction factor APOBEC3A is a member of the APOBEC3 family of DNA cytidine deaminases, which restrict HIV-1 replication by hypermutating viral cDNA and inhibiting reverse transcription and integration^41^. APOBEC3A has also been recently highlighted in the maintenance of latency in HIV-1 infected CD4^+^T cells^15^.

It has been proposed that differentiation of monocytes into MDM reduces expression of innate restriction factors, rendering the otherwise relatively refractory monocytes into an HIV-1 permissive MDM phenotype^16^. In this study, the dramatic increase in APOBEC3A gene expression and the corresponding protein synthesis of APOBEC3A in MDM that were treated with ALFQ, coincident with the ability of those MDM to restrict the HIV-1 infectivity suggest that ALFQ renders an HIV-1-permissive target into a more restrictive phenotype. Experiments with A3A siRNA and Western blotting demonstrated a significant decrease in APOBEC3A gene and protein expression with a concomitant loss of restriction of HIV-1 infectivity. Thus, our study demonstrates that the induction of APOBEC3A by ALFQ restricts HIV-1 infection in ALFQ-treated MDM. Interestingly, donor #118, showed enhanced gene and protein expression of APOBEC3A and IFI16, and decreased levels of pSAMHD1. These outcomes occur in the proinflammatory cytokine environment induced with ALFQ treatment. Although our study did not address the relationship between the inflammatory environment and synthesis of innate anti-HIV-1 proteins, a recent study^14^ showed that the presence of APOBEC3A was necessary for induction of proinflammatory cytokines and upregulation of CD86 expression in macrophages. Our study also showed increased levels of proinflammatory cytokines, MHC-II and CD86 expression coincident with APOBEC3A synthesis in ALFQ-exposed MDM. Therefore it may be plausible to postulate that ALFQ induces the synthesis of APOBEC3A and possibly other anti-viral proteins which promote the induction of a proinflammatory environment that polarize MDM into an M1 phenotype, rendering them less permissive to HIV-1 infection^42,43^. This indicates a possibility that the outcome of the HIV-1/target cell relationship could be tilted in favor of the target cell.

ALFQ as a potent adjuvant has shown great promise in several preclinical studies with parasitic (malaria), viral (HIV and SARS-CoV-2), and bacterial (Campylobacter) vaccine formulations^33,44^. Three ongoing phase 1 clinical trials which utilize ALFQ as the adjuvant have been recently completed. Two of these were with circumsporozoite malaria proteins (ClinicalTrials.gov Identifier: NCT04296279 and NCT04268420) and the third one was with SARS-CoV-2 spike-ferritin nanoparticle (ClinicalTrials.gov Identifier: NCT04784767). ALFQ will also be used in an ongoing recent phase 1 trial in Bangkok, Thailand with HIV envelope protein (NCT04658667). In addition to the use of ALFQ in HIV-1 vaccine studies, our data also highlights the potential use of ALFQ as a therapeutic agent since it targets the innate immune system.

## Materials and methods

### Flow cytometry reagents

The following human monoclonal antibodies (mAbs) anti CD11b PE (clone ICRF44), CD11b FITC (clone ICRF44), CD14 PerCP (clone MoP9), CD4 PE (clone RPA-T4), CD3 PerCP (clone SK7), CD80 PE (L307.4), CD86 APC (2331 (FUN-1)), CD16 FITC (clone 3G8), CD32 APC (clone 8.26) were obtained from BD Pharmingen. Anti-CD169 APC (clone 7–239), CD195 FITC (clone HEK/1/85a), HLA-A,B,C PE (clone W6/32), HLA-DR, DP, DQ FITC (clone Tu39), FITC Annexin V Apoptosis kit with 7AAD were obtained from BioLegend. Quantum Simply Cellular anti-mouse IgG microbeads were obtained from Bangs Laboratories. Anti-p24-FITC and anti-p24-RD1 were purchased from Beckman Coulter. Fixation and permeabilization buffers (Reagents A and B) were from Caltag.

### Media and reagents

Media components and reagents were obtained as follows: RPMI-1640 (Biowhittaker), L-glutamine and penicillin/streptomycin (Quality Biologicals Inc.), Accutase (eBiosciences), recombinant human M-CSF (Peprotech), polybrene (Sigma-Aldrich) and fetal bovine serum (Gemini Bio Products). Attachment media (RPMI-1640 supplemented with 1% L-glutamine, and 1% penicillin/streptomycin), Monocyte media (RPMI-1640 supplemented with 10% heat-inactivated FBS, 1% L-glutamine, and 1% penicillin/streptomycin), Infection media (monocyte media containing polybrene at 2 μg/ml), and M-CSF media (monocyte media supplemented with 50 ng/ml M-CSF) were used for the various experiments. Dimyristoyl phosphatidylcholine (DMPC), dimyristoyl phosphatidylglycerol (DMPG), cholesterol, and synthetic MPLA (3D-PHAD®), were purchased from Avanti Polar Lipids (Alabaster, AL, USA). DMPC and cholesterol were dissolved in freshly distilled chloroform and DMPG and 3D-PHAD® were dissolved in chloroform:methanol (9:1). Purified QS-21 (Desert King International San Diego, CA, USA) was dissolved in Sorensen’s PBS, pH 6.2 at 1 mg/ml.

### Virus purification

HIV-1 subtype B primary virus, US-1, was grown in PBMCs from the virus stock provided by Dr. Victoria Polonis (USMHRP). The primary virus was purified as previously described^45^. Infectivity and p24 concentrations were determined before and after purification to ensure that infectivity was not lost during the purification procedure.

### Adjuvant formulations

Adjuvant formulations (Table 1) were prepared as described previously^31^. Army Liposome Formulation (ALF55 liposomes) containing DMPC, DMPG, cholesterol, and a synthetic MPLA (3D-PHAD®, Avanti Polar Lipids, AL), was prepared by the lipid deposition technique as previously described^46^. ALF55 liposomes are composed of DMPC:DMPG:Cholesterol:3D-PHAD® in a molar ratio of 9:1:12.2:0.114, resulting in a liposomal cholesterol concentration of 55 mol% relative to the structural phospholipids DMPC and DMPG. To generate ALF55, lipid stock solutions were combined, dried via rotary evaporation, and stored under vacuum for at least 6 h to evaporate residual solvents. The ALF55 lipid film was rehydrated in Sorenson’s PBS, pH 6.2 for a final MPLA:phospholipid ratio of 1:88, and microfluidized to generate SUV liposomes. To prepare Army Liposome Formulation containing QS-21 (ALFQ), aqueous QS-21 was added to ALF55 liposomes for a final 3D-PHAD® concentration of 200 μg/ml, QS-21 concentration of 100 μg/ml, and a molar ratio of 9:1:12.2:0.1136:0.0439 (DMPC:DMPG:Cholesterol:3D-PHAD®:QS-21). QS-21 irreversibly binds to cholesterol in the membrane of the ALF55 liposomes under these conditions with no detectable free QS-21 present in the buffer^47^. All formulations were stored at 4 °C prior to use.

### Microfluidization of ALFQ to generate small unilamellar vesicle ALFQ (ALFQsuv)

ALFQsuvs were prepared using a Microfluidizer (LV1, Microfluidics). One ml of ALFQ (lipid concentration of 28.325 mM) was diluted 10 times with Sorensen’s phosphate-buffered saline (SPBS). The microfluidizer was prepared by injecting 6 ml of 70% isopropanol thrice and 6 ml of SPBS five times at 30,000 psi. This was followed by the passage of 6 ml of the above diluted ALFQ sample through the microfluidizer at 30,000 psi. The process was repeated six times. A gel loading pipette tip was used to remove residual liquid from the intake and output ports of the microfluidizer. Finally, the microfluidized ALFQ was filter-sterilized through a 0.22 µm filter and stored at 4℃ until use. The concentration of liposomes was determined by the cholesterol assay^48^.

### Cryo-electron microscopy

Cryo-electron microscopy was performed under a contract with University of Virginia (UVA). Briefly liposome samples (~ 3.5 μl) were applied to a glow-discharged, perforated carbon-coated grid (2/1-3C C-Flat; Protochips, Raleigh, NC), manually blotted to near dryness with filter paper, and rapidly plunged into liquid ethane to freeze the sample. The grids were stored in liquid nitrogen, then transferred to a Gatan 626 cryo-specimen holder (Gatan, Warrrendale, PA) and maintained at ~ 180 °C. Low-dose images were collected on a Tecnai F20 Twin transmission electron microscope (FEI {now ThermoFisher Scientific}, Hillsboro, OR) operating at 120 kV^49^. The digital micrographs were recorded on a Gatan US4000 CCD or a Teitz XF416 camera.

### Dynamic light scattering (DLS)

The hydrodynamic diameter for ALF55, ALFQ, and ALFQsuv at a 3D-PHAD® concentration of 100 µg/ml was determined by intensity of DLS using a Malvern Zetasizer Nano S (Malvern, Worcestershire, UK) equipped with a 633 nm laser.

### In vitro differentiation of MDM

Peripheral blood mononuclear cells (PBMCs) were isolated by Ficoll density gradient centrifugation from healthy HIV-1 seronegative donors under an internal review board (IRB)-approved protocol, RV229B (WRAIR Protocol #1386). This protocol “Apheresis of blood components from healthy volunteers for in vitro research” and all related documents were approved by the following independent Institutional Review Boards (IRBs): Division of Human Subject Protection, Walter Reed Army Institute of Research; Ethical Review Committee for Research in Human Subjects. Informed consent was obtained from all participants. All experiments were performed in accordance with relevant guidelines and regulations. Monocytes were enriched from the PBMCs by plastic adherence in 24-well plates and differentiated into monocyte-derived macrophages (MDM) in M-CSF media, as previously described^45^. MDM were used on day 5 post-culture.

### Generation of adjuvant-derived supernatants

On day 4 post-culture, ALF55 or ALFQ were added to cultures of MDM (3–4 × 10^5^cells/well) and the cultures were incubated at 37 °C/5%CO_2_ for 24 h, after which the cells and supernatants were harvested. MDM cultured in media not exposed to any adjuvant served as controls. In each appropriate well, the final concentration of 3D-PHAD® was 0.5 μg/ml and QS-21 was 0.25 μg/ml. Cells and supernatants were harvested. The viability of the cells was determined by trypan blue exclusion as well as Annexin V/7AAD staining. Supernatants were clarified twice at 13,200 rcf for 20 min at 4 °C and transferred into clean tubes. Samples were stored at − 20 °C for subsequent analyses and assays.

### Cytokine and chemokine assays

Cytokines and chemokines (GM-CSF, IFN-γ, IL-10, IL-12p70, IL-18, IL-1β, IL-23, IL-27, IL-6, IL-8, TNF-α, IP-10, MCP-1, MIP-1α and MIP-1β) were quantified using a custom-made U-Plex electrochemiluminescent immunoassay (Meso Scale Discovery, Rockville, MD, US). Cells were stimulated as previously described and the culture supernatants were harvested. Assay was run according to the instructions of the manufacturer for U-Plex custom kits. Duplicate measurements were obtained for both samples and standards. Data was collected using the MESO QuickPlex SQ 120 instrument and the MSD Discovery Workbench software.

### Detection of cell surface molecules

Cells were washed in cold FACS buffer (PBS-containing 0.5% bovine serum albumin (BSA)) and blocked in FACS buffer containing 10% normal goat serum. Following incubation for 20 min at 4 °C with a cocktail containing 5-10 μg of specific mAb, the cells were washed in cold FACS buffer, fixed in PBS-containing 2% paraformaldehyde, and subsequently acquired on a FACSCalibur (BD Biosciences, San Jose, CA). Data analyses were performed on gated CD14^+^ cells, using FlowJo 8.8.6 software (TreeStar Inc., Ashland, OR).

### Quantitation of cell surface receptors

The number of receptors/cell was determined using Quantum Simply Cellular Beads (Bangs Laboratories Inc., IN, USA) according to the manufacturer’s instructions, and as previously described^25^. Briefly, MDM (0.5 × 10^6^/tube) were preincubated with 10% normal goat sera, followed by the addition of a mAb cocktail for 30 min on ice. Cells were then fixed with 2% formalin. Beads from the kit were stained with anti-human HLA-A, B, C-FITC, anti-human HLA-DR, DP, DQ-FITC, anti-human CD80-PE, anti-human CD86-APC, anti-human CD16-FITC, anti-human CD32-PE, anti-human CD169-APC. Individual standard curves were established using the stained beads. Samples were acquired on a FACSCalibur and analyzed with FlowJo 8.8.6 software. Data were placed into QuickCal version 2.3 software (Bangs Laboratories), and the number of receptors/cell was extrapolated from the standard curves generated with the stained beads.

### HIV-1 infection of MDM

MDM (3–4 × 10^5^cells/well) were pre-treated with ALF55, ALFQ or ALFQsuv for 24 h at 37 °C/5%CO_2_. MDM not exposed to any adjuvant served as controls. Cells were gently rinsed, and 300 μl infection media containing 3 ng HIV-1 p24 (US-1) was added to each well. The cultures were spinoculated at 1500 rpm for 2 h at 25 °C-30°C, and further incubated at 37 °C/5%CO_2_ for 1 h. Unadsorbed virus was removed, and 1 ml M-CSF media containing polybrene at 2 μg/ml was added to each well. The plates were further incubated at 37 °C/5%CO_2_ for 4–7 days. MDM were harvested and the presence of intracellular p24, indicative of HIV-1 infection, was determined by flow cytometry. The MDM were also analyzed for evidence of Annexin V positive cells, indicative of apoptosis, by flow cytometry.

### Detection of intracellular p24

Staining for detection of intracellular p24 in HIV-1 infected MDM was carried out as previously described^25^. Data analyses were performed using FlowJo 8.8.6 software (TreeStar Inc., Ashland, OR).

### HIV-1 capture and entry assay

MDM (3–4 × 10^5^cells/well) were pre-incubated for 24 h at 37 °C/5%CO_2_ with ALF55 or ALFQ. MDM not exposed to any adjuvant served as controls. Cells were gently rinsed, and 300 μl infection media containing 3 ng HIV-1 p24 (US-1) was added to each well. The cultures were spinoculated at 1500 rpm for 2 h at 25–30 °C. MDM were harvested and washed (3×) in PBS. Cells were lysed and RNA was isolated using the RNeasy Mini Plus Kit (Qiagen). The samples were evaluated for the presence of gag RNA by qRT-PCR to determine the amount of virus captured by MDM^27^.

### Quantitation of HIV-1 DNA

MDM (3–4 × 10^5^cells/well) were pre-incubated for 24 h at 37 °C/5%CO_2_ with ALF55 or ALFQ. MDM not exposed to any adjuvant served as controls. Cells were gently rinsed, and 300 µl infection media containing 3 ng HIV-1 p24 (US-1) was added to each well. The cultures were spinoculated at 1500 rpm for 2 h at 25–30 °C. MDM were harvested and washed (3×) in PBS. DNA was extracted using the DNeasy Blood and Tissue kit (Qiagen). Cells were evaluated for the presence of HIV-1 strong stop and GAPDH DNA by qPCR with the Taqman 2X Gene Expression Master Mix (Applied Biosystems) as previously described^27^.

### Analysis of cellular restriction factor genes

Cellular RNA was isolated using the RNeasy Plus Mini Kit (Qiagen). The RNA was eluted in RNase free water, and the concentration was determined with a Nanodrop 2000 (Thermo Scientific). The qRT-PCR reactions were performed using the TaqMan RNA-to-Ct Master Mix and the Viia7. Reactions (50 µl) were performed in the presence of the master mix, and the following predesigned Human TaqMan assays: APOBEC3A_B (Hs00377444_m1), APOBEC3D (Hs00537163_m1), APOBEC3F (Hs00736570_m1), APOBEC3G (Hs00222415_m1), TRIM5 (Hs01552558_m1), TRIM22 (Hs00232319_m1), SAMDH1 (Hs00210019), and IFI16 (Hs00194261) (Thermo Scientific). Cycling parameters were 48 °C for 20 min, 95 °C for 10 min, then, 45 cycles at 95 °C for 15 s, and 59 °C for 1 min. First, the triplicate samples were pooled together for screening of changes in gene expression of the above genes using 50 ng of RNA in each qRT-PCR reaction. The pooled samples that demonstrated a change in gene expression were further evaluated and each individual sample was examined for gene expression using 25 ng of RNA in each qRT-PCR reaction. DCt values were calculated to normalize the Ct value of the gene of interest as a function of the Ct value of the GAPDH RNA signal. Then fold-change in gene expression was determined by calculating the 2^(-DDCt) for the various conditions in relation to the control (no adjuvant).

### Transfection of siRNA

MDM were transfected with equimolar mix of two human APOBEC3A siRNAs (Silencer 45715 and Silencer 45810, Life Technologies) or 100 nM of negative control siRNA (AM4611, Life Technologies) using Lipofectamine RNAiMax (Life Technologies), according to the manufacturer’s instructions, 24 h before treatment with ALFQ. Cells were washed and cultured in media with ALFQ for 24 h. Cells were harvested and APOBEC3A gene expression was analyzed by qRT-PCR and APOBEC3A protein by Western blotting as described below. For determination of HIV-1 infection, non-transfected or siRNA transfected cultures were treated with ALFQ for 24 h and subsequently infected with HIV-1 for 5 days. The cells were harvested and the presence of intracellular p24, indicative of HIV-1 infection, was determined by flow cytometry.

### SDS Polyacrylamide gel electrophoresis and Western blotting

Briefly, MDM cell pellets that had not been exposed to adjuvant or were exposed to ALF55 or ALFQ were incubated with Mammalian Protein Extraction Reagent, M-PER (ThermoFisher Scientific, CA) with Protease inhibitor cocktail (Sigma-Aldrich, MO) for 10 min at room temperature (RT). The lysates were centrifuged at 14,000 × g for 15 min to remove the cell debris and the protein concentrations quantitated using a Nanodrop Lite Spectrophotometer (ThermoFisher Scientific, CA). The lysates were normalized to protein concentration (33 µg per sample) and mixed with Laemmli buffer (at an approximate ratio of 1:3 sample to Laemmli buffer). The resulting sample mix was heated at 95 °C under vortexing (at 1000 rpm) for 3 min and subsequently spun down for 10 s. A total sample mix volume of 20 µl was loaded onto each lane of a Novex™ WedgeWell™ 4–20%, Tris–Glycine Gel (Invitrogen, CA). Transfer of proteins to a PVDF membrane was performed at 20 V (1 min), 23 V (4 min), and 25 V (2 min) using the iBlot2 Western Blotting System (Thermo Fisher Scientific, CA) and iBlot™ 2 Transfer Stacks (Invitrogen, CA). Membranes were blocked with 5% skim milk in PBS containing 0.1% Tween 20 (PBS-T) for 30 min at 4 °C, and then incubated overnight at 4 °C with primary antibody at a 1:1000 dilution in PBS-T. Antibodies for protein targets include rabbit anti-APOBEC3A (Novus Biologicals), rabbit anti-APOBEC3A (Thermo Fisher), rabbit anti-IFI16 (Abcam), rabbit anti-SAMHD-1 (Abcam), rabbit anti-Phospho-SAMHD-1 (Thr592) (Cell Signaling) and rabbit anti-GAPDH (Abcam). Rabbit anti-APOBEC3A (Novus Biologicals) was used with lysates of donors #170 and #118, whereas rabbit anti-APOBEC3A (Thermo Fisher) was used for donors #205 and #008. (anti-APOBEC3A, Thermo Fisher). The use of a different source for anti-APOBEC3A antibody was due to supply chain difficulties. The membranes were subsequently washed for 5 min (three times) in PBS-T at room temperature (RM), and then incubated in 1 × PBS with goat anti-rabbit HRP secondary antibody (System Biosciences, CA) at RT for 1 h. The membranes were then washed for 5 min in PBS-T (twice) and with PBS (once) prior to developing using Clarity (Bio-Rad, CA) and/or Clarity Max ECL Western Blotting Substrates (Bio-Rad, CA). Chemiluminescence was imaged using a ChemiDoc Touch Imaging System (BIO-RAD) and images processed using Image Lab (Software version 6.0.1.0; Bio-Rad, CA). Densitometry analysis was used to measure the signal from each protein band and analyzed using ImageJ software (National Institutes of Health; Bethesda, MD). The signal density counts were obtained for each band, subtracting background signal, using the same capture area for all bands. Each protein band signal was normalized to its corresponding GAPDH band to account for potential variations between samples.

### Statistical analysis

Statistical methods used are detailed in the figure legends. Cytokine responses were compared by two-tailed Student t-test. Cellular anti-viral genes were compared by two-tailed Mann–Whitney test. Group means of experimental data for number of cell receptors, percentage of HIV-1 infected MDM, apoptotic MDM and HIV-1 RNA and DNA copies were compared by One-way ANOVA. Data are displayed as scatter dot plots or bar graphs with mean ± standard deviation. No data point was excluded from the analyses. p-values less than or equal to 0.05 were considered statistically significant. Analyses were carried out with GraphPad Prism 7 (version 5.0c; GraphPad Software (La Jolla, CA, USA).

### Ethics statement

RV229B (WRAIR Protocol #1386): This protocol “Apheresis of blood components from healthy volunteers for in vitro research” and all related documents were approved by the following independent Institutional Review Boards (IRBs): Division of Human Subject Protection, Walter Reed Army Institute of Research; Ethical Review Committee for Research in Human Subjects. All volunteers provided written informed consent following discussion and counseling by the clinical study team prior to enrollment and before the blood draw.

### Disclaimer

The views expressed are those of the authors and should not be construed to represent the positions of the U.S. Army or the Department of Defense.

## Supplementary Information

Supplementary Information.
