# Supplementary material for: Army liposome formulation containing QS-21 render human monocyte-derived macrophages less permissive to HIV-1 infection by upregulating APOBEC3A
Source: Sci Rep. 2022 May 9;12:7570. doi: 10.1038/s41598-022-11230-8 (PMC9082986; doi:10.1038/s41598-022-11230-8)
Supplement: Supplementary file 1 — Supplementary Information. [file 41598_2022_11230_MOESM1_ESM.pptx]

## Slide 1
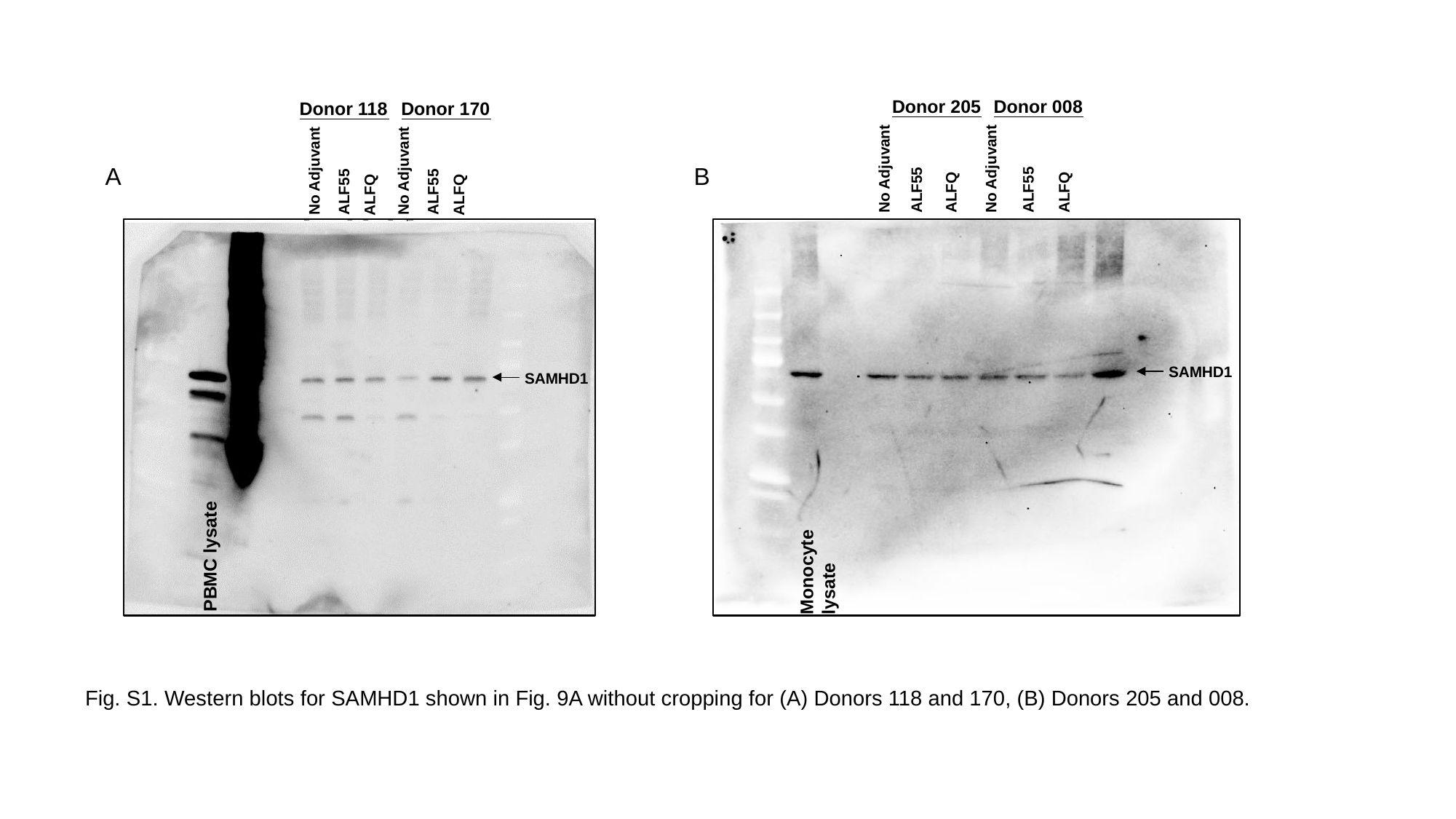

Donor 205
Donor 008
Donor 118
Donor 170
No Adjuvant
No Adjuvant
No Adjuvant
No Adjuvant
A
B
ALF55
ALF55
ALFQ
ALFQ
ALF55
ALF55
ALFQ
ALFQ
SAMHD1
SAMHD1
Monocyte lysate
PBMC lysate
Fig. S1. Western blots for SAMHD1 shown in Fig. 9A without cropping for (A) Donors 118 and 170, (B) Donors 205 and 008.

## Slide 2
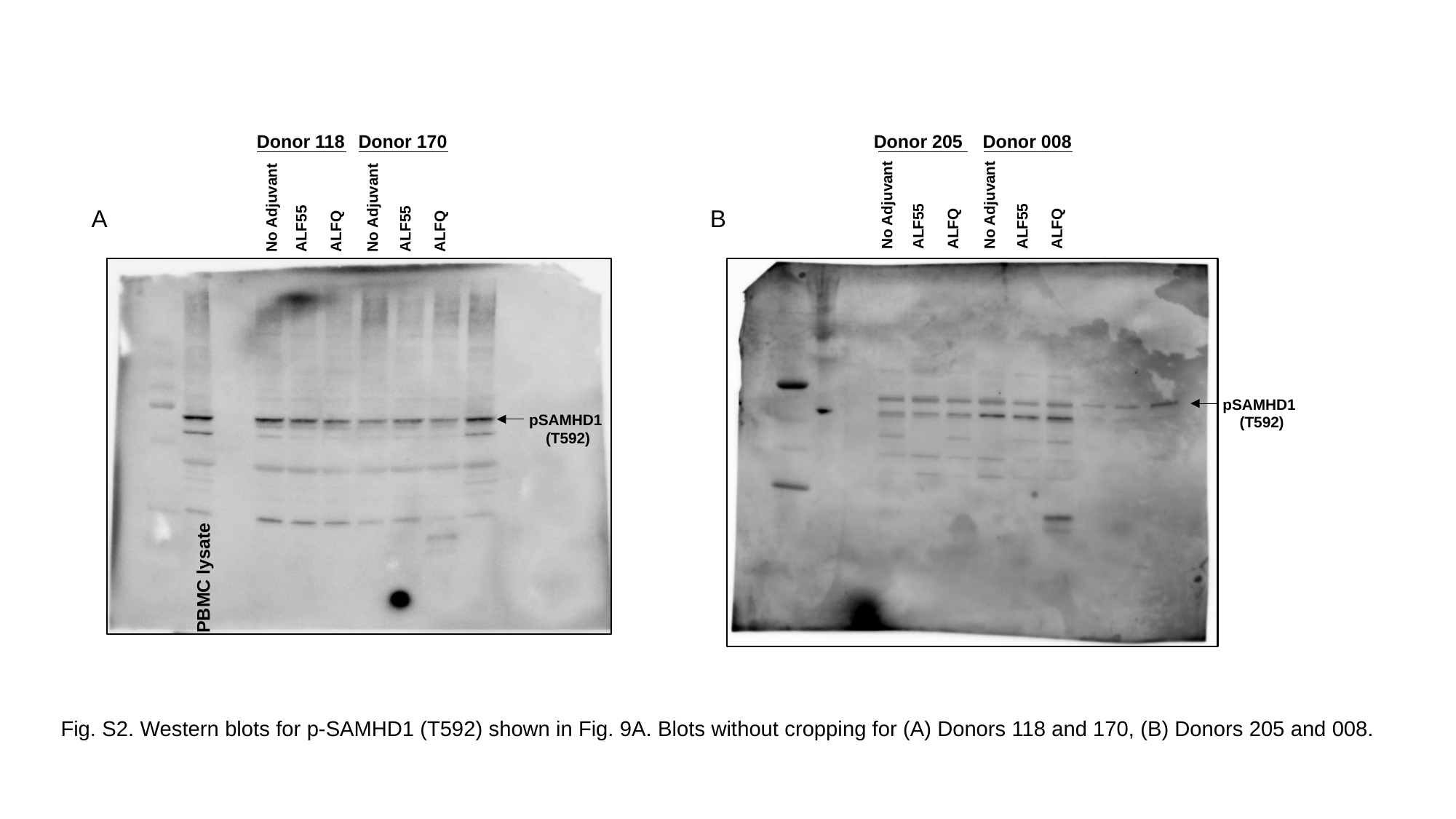

Donor 118
Donor 170
Donor 205
Donor 008
No Adjuvant
No Adjuvant
No Adjuvant
No Adjuvant
A
B
ALF55
ALF55
ALFQ
ALFQ
ALF55
ALF55
ALFQ
ALFQ
pSAMHD1
 (T592)
pSAMHD1
 (T592)
PBMC lysate
Fig. S2. Western blots for p-SAMHD1 (T592) shown in Fig. 9A. Blots without cropping for (A) Donors 118 and 170, (B) Donors 205 and 008.

## Slide 3
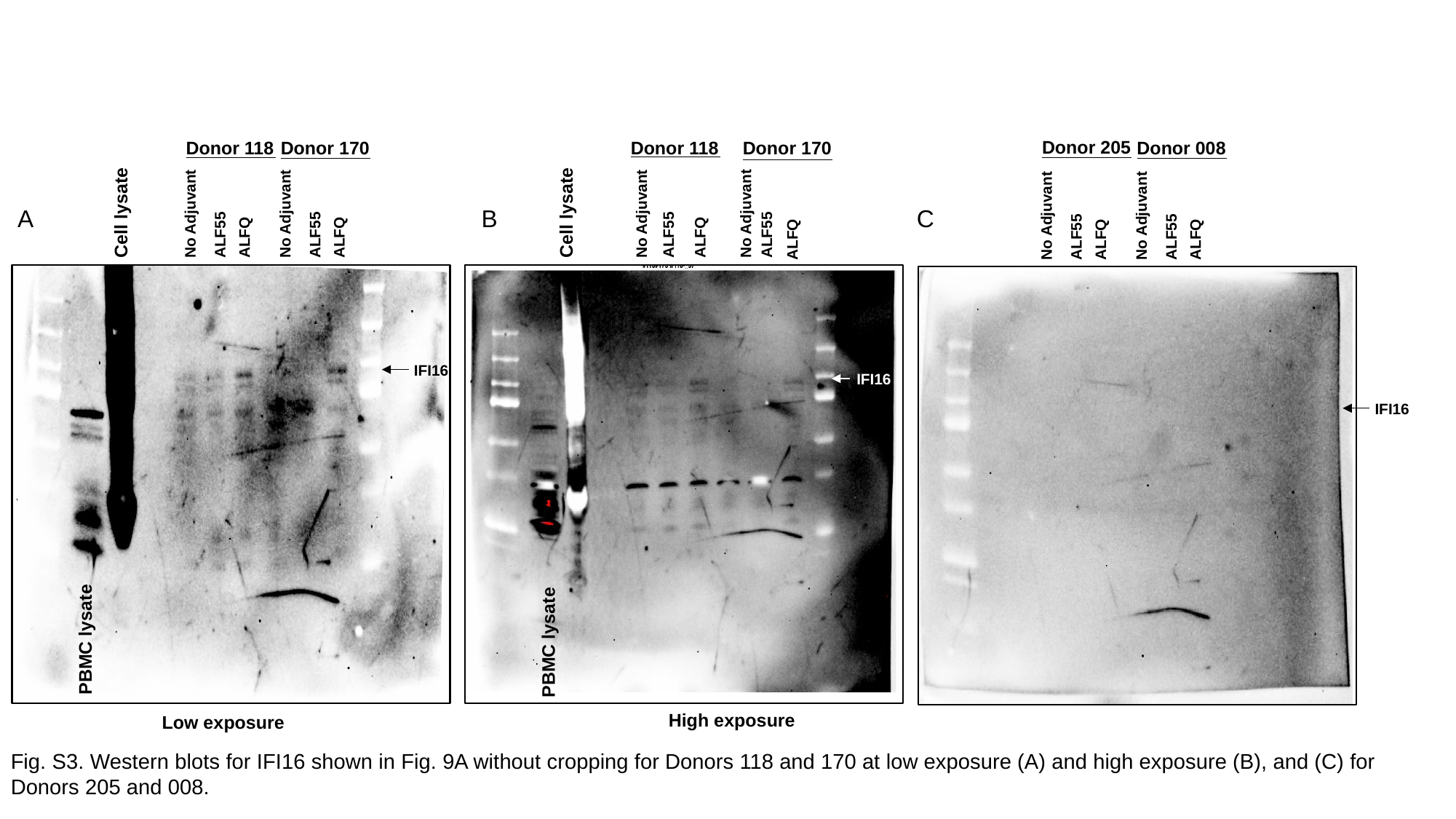

Donor 205
Donor 008
Donor 118
Donor 170
Donor 118
Donor 170
Cell lysate
Cell lysate
No Adjuvant
No Adjuvant
No Adjuvant
No Adjuvant
No Adjuvant
No Adjuvant
A
B
C
ALF55
ALF55
ALF55
ALF55
ALFQ
ALFQ
ALFQ
ALF55
ALF55
ALFQ
ALFQ
ALFQ
IFI16
IFI16
IFI16
PBMC lysate
PBMC lysate
High exposure
Low exposure
Fig. S3. Western blots for IFI16 shown in Fig. 9A without cropping for Donors 118 and 170 at low exposure (A) and high exposure (B), and (C) for Donors 205 and 008.

## Slide 4
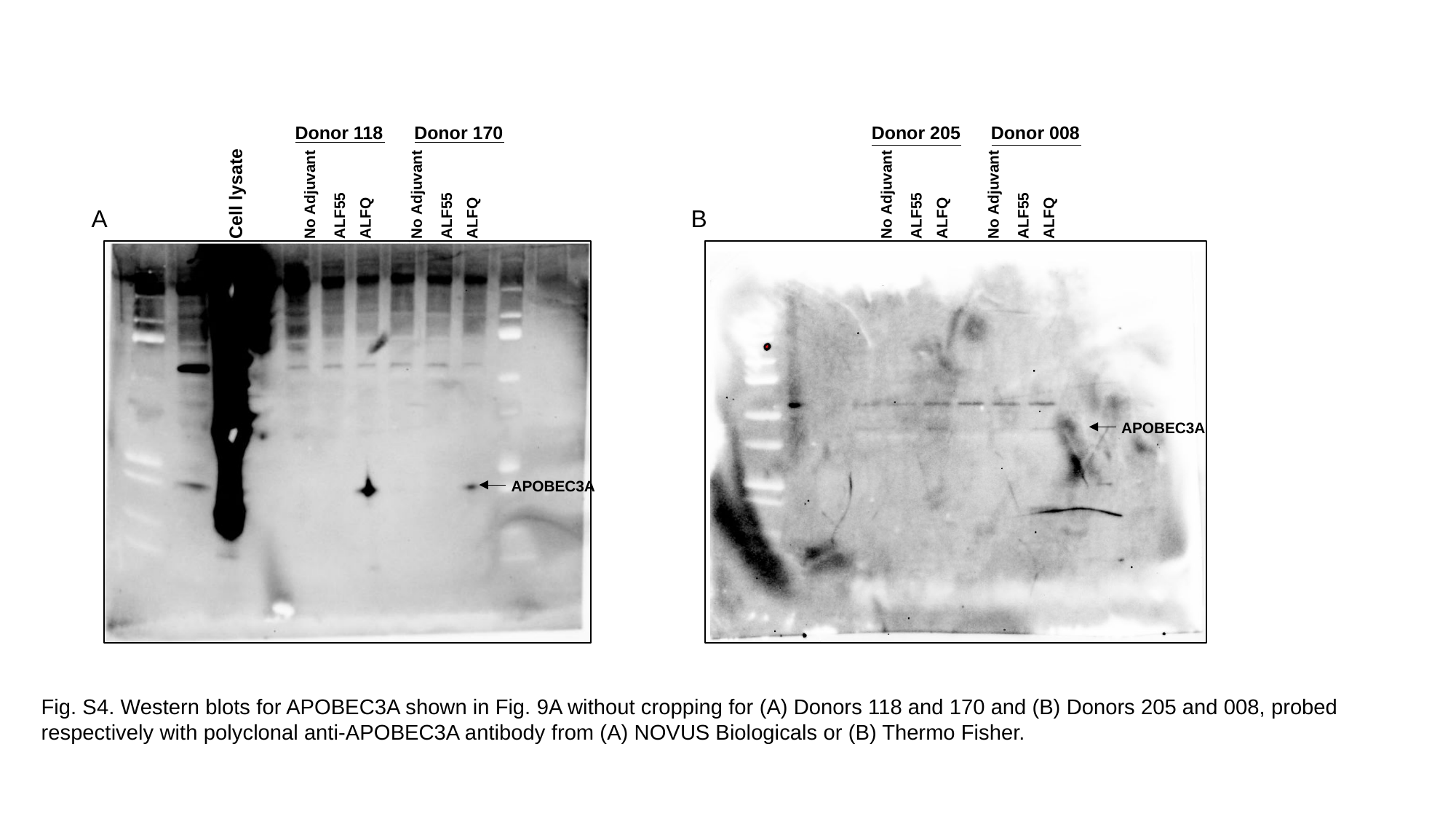

Donor 118
Donor 170
Donor 205
Donor 008
Cell lysate
No Adjuvant
No Adjuvant
No Adjuvant
No Adjuvant
A
B
ALF55
ALF55
ALF55
ALF55
ALFQ
ALFQ
ALFQ
ALFQ
APOBEC3A
APOBEC3A
Fig. S4. Western blots for APOBEC3A shown in Fig. 9A without cropping for (A) Donors 118 and 170 and (B) Donors 205 and 008, probed respectively with polyclonal anti-APOBEC3A antibody from (A) NOVUS Biologicals or (B) Thermo Fisher.

## Slide 5
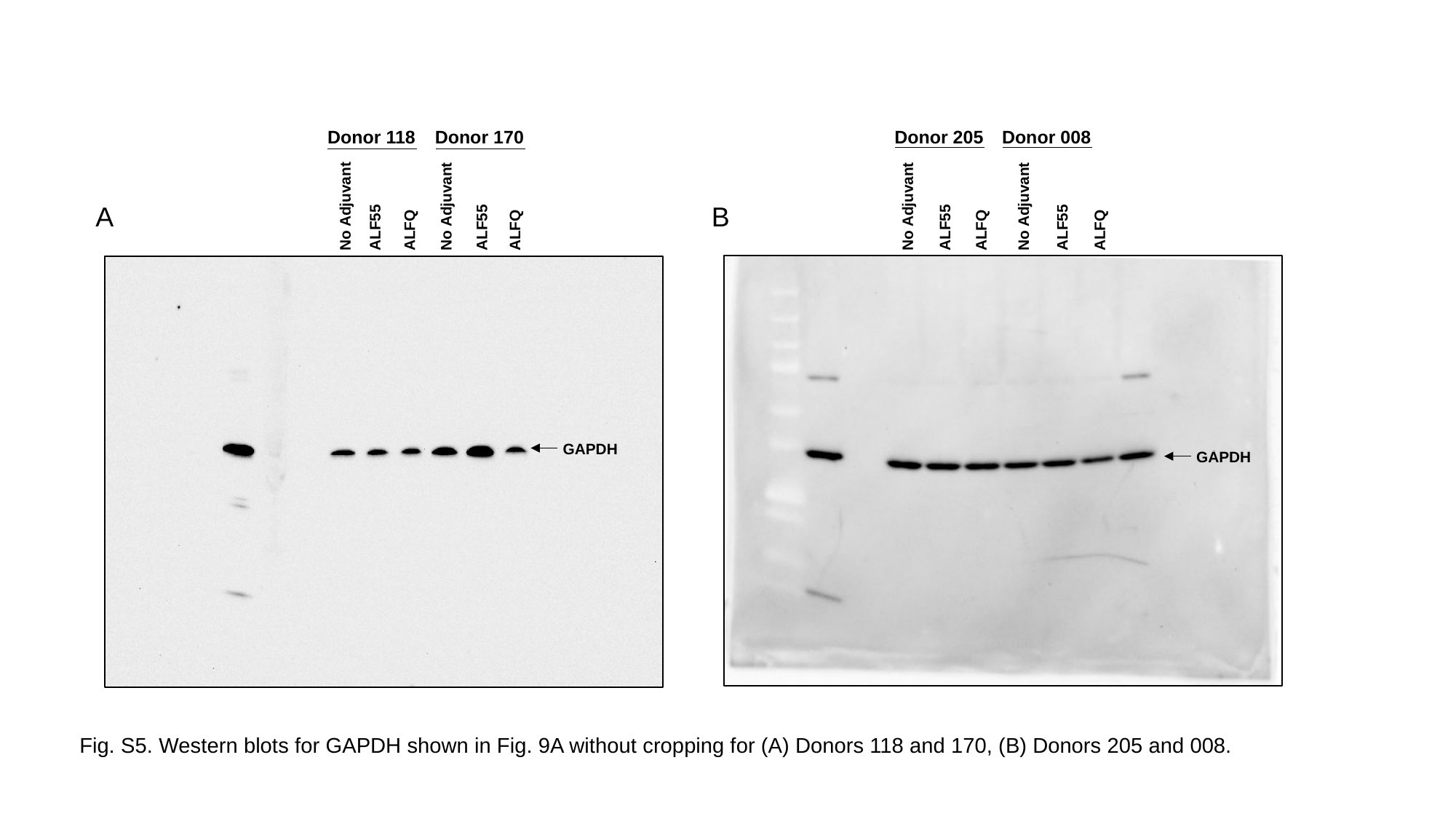

Donor 118
Donor 170
Donor 205
Donor 008
No Adjuvant
No Adjuvant
No Adjuvant
No Adjuvant
A
B
ALF55
ALF55
ALF55
ALF55
ALFQ
ALFQ
ALFQ
ALFQ
GAPDH
GAPDH
Fig. S5. Western blots for GAPDH shown in Fig. 9A without cropping for (A) Donors 118 and 170, (B) Donors 205 and 008.

## Slide 6
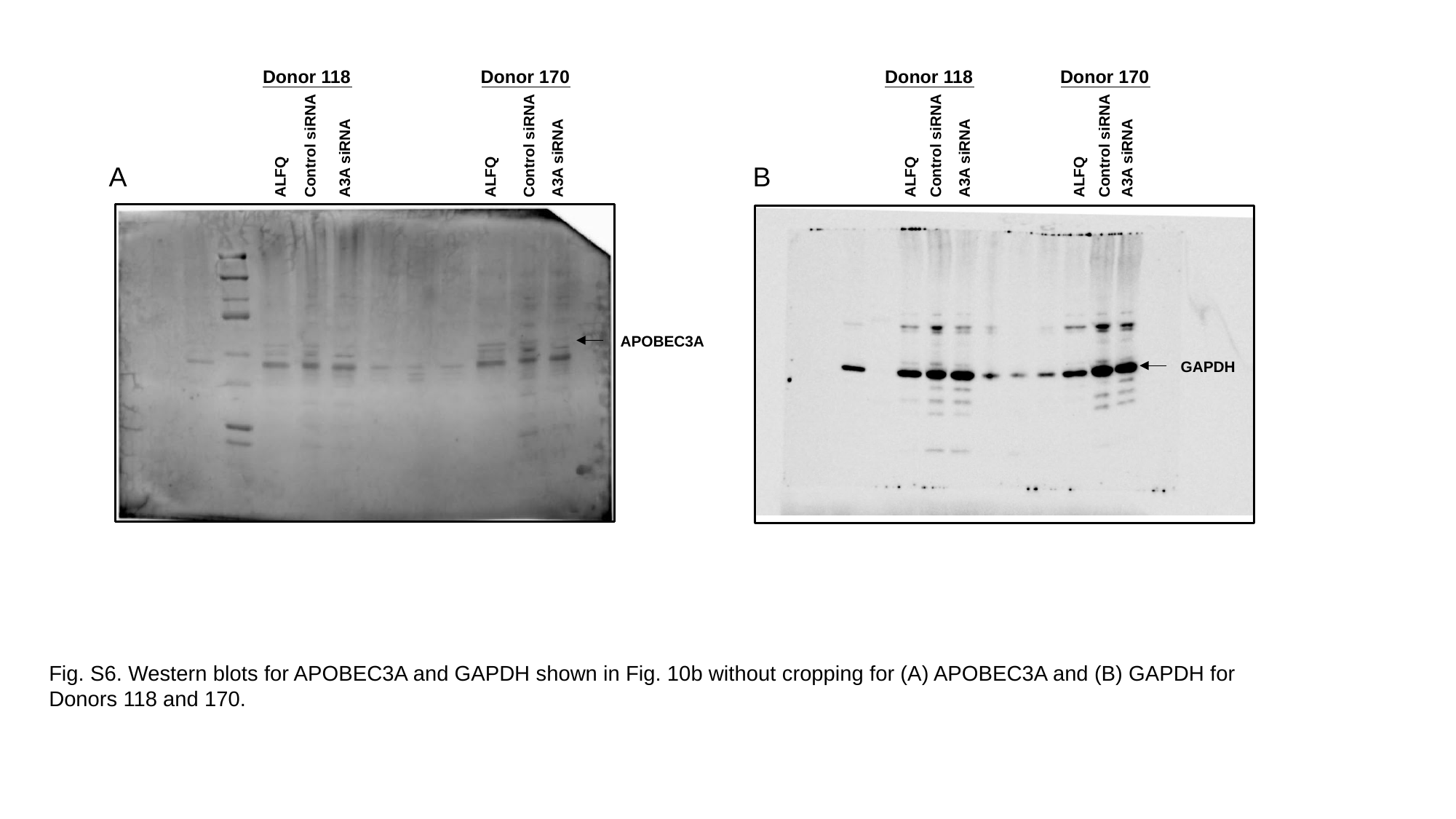

Donor 118
Donor 170
Donor 118
Donor 170
Control siRNA
Control siRNA
Control siRNA
Control siRNA
A3A siRNA
A3A siRNA
A3A siRNA
A3A siRNA
A
B
ALFQ
ALFQ
ALFQ
ALFQ
APOBEC3A
GAPDH
Fig. S6. Western blots for APOBEC3A and GAPDH shown in Fig. 10b without cropping for (A) APOBEC3A and (B) GAPDH for
Donors 118 and 170.
